# Analytical prediction of electromagnetic performance for surface-embedded permanent magnet in-wheel machines considering iron’s nonlinearity

**DOI:** 10.1038/s41598-024-77261-5

**Published:** 2024-11-01

**Authors:** Heshan Zhang, Mengwei Fan, Jie Qiao, Xianjin He, Minglei Yang, Jiying Tuo

**Affiliations:** 1 College of Traffic & Transportation, Chongqing Jiaotong University, Chongqing, 400074 China; 2 China Automotive Engineering Research Institute Co., Ltd, Chongqing, 401122 China; 3 School of Automotive Studies, Tongji University, Shanghai, 200092 China; 4 College of Vehicle Engineering, Chongqing University of Technology, Chongqing, 400054 China

**Keywords:** In-wheel machine, Magnetic field, Permeability, Analytical model, Magnetic saturation, Torque, Electrical and electronic engineering, Mechanical engineering

## Abstract

Accurate magnetic field calculation is the premise of electromagnetic performance prediction. Conventional subdomain (SD) techniques assume that the iron’s relative permeability is infinite, leading to falsely overestimated flux density. We propose an accurate magnetic field analytical model for permanent magnet (PM) in-wheel machines considering iron’s magnetization nonlinearity and saturation. Specifically, according to the excitation source and topology, the entire solution domain of the machine is divided into sub-regions such as stator slots/teeth, stator slot-openings/tooth-tips, air-gap, and rotor slots/teeth, etc. Poisson’s or Laplace’s magnetic vector potential (MVP) equations are solved using Maxwell’s electromagnetic theory and complex Fourier series methods in each sub-region. Specifically, in our approach, The Cauchy product theorem addresses the discontinuous magnetic permeability change at the slot and tooth interface. The machine’s magnetic saturation effect is considered by combining the actual magnetization characteristics of iron with an iterative algorithm. The general solution for the MVP is solved using the boundary conditions between adjacent subregions. Subsequently, electromagnetic properties such as air-gap flux density, back electromotive force (EMF), and electromagnetic torque are obtained. The accuracy of the analytical model is verified by finite element analysis (FEA) and prototype tests, which proved that the proposed analytical model can consider the iron’s nonlinearity and the magnetic saturation. In addition, the inaccurate overestimation of electromagnetic torque and air-gap magnetic flux density by the conventional SD techniques has also been proven.

**Supplementary Information:**

The online version contains supplementary material available at 10.1038/s41598-024-77261-5.

## Introduction

Multi-wheel individually driven electric vehicles (EVs) have become a new development orientation in new energy vehicles due to their high transmission efficiency and control flexibility^1–3^. The in-wheel machine integrates the machine, transmission mechanism, brake, etc. The driving power is directly transmitted to the wheels through the machine rotor, eliminating the traditional mechanical connections, simplifying the transmission system’s structure, improving the EVs’ response speed and control accuracy, and facilitating the realization of mechatronics^4–6^.

Permanent magnet (PM) machines have the advantages of high power density, high efficiency, long life, etc. So, it is widely used as a traction machine for EVs^7,8^. The outer rotor PM machine is usually used as the direct-drive in-wheel machine. The magnets are typically surface-mounted or surface-embedded topology to reduce the mass and volume of the rotor. However, the latter has many advantages over the former. i.e., the surface-embedded magnet topology can possess reluctance torque, reduce eddy current loss, have better magnetic weakening ability, and have higher mechanical strength^9,10^. Therefore, the in-wheel machine studied in this paper adopts a topology of outer rotor with embedded magnets on the surface. In order to meet the EVs’ operating requirements of starting, climbing, accelerating, the in-wheel machine often operates under high load conditions, producing magnetic saturation effects to suppress the electromagnetic torque. When the machine generates a magnetic saturation effect, the magnetic permeability of the stator and rotor cores decreases, leading to a decrease in air-gap flux density, which in turn causes attenuation of electromagnetic torque.

Accurately calculating the magnetic field distribution of the in-wheel machine is the prerequisite for obtaining electromagnetic performance. Compared to the two-dimensional (2-D) finite element analysis (FEA), the analytical method significantly shortens the calculation time while ensuring accuracy. Furthermore, it can theoretically clarify the essential relationship between electromagnetic performances and design quantities. Therefore, the initial design and optimization stages are performed using analytical methods, and then FEA is used to validate and refine the analytical predictions. Commonly used analytical methods include equivalent magnetic circuit (EMC)^11,12^, conformal transformation (CT)^13,14^, and sub-domain (SD) technique^15,16^. The EMC can only calculate the average flux density but cannot consider the magnetic field harmonics. The CT thinks stator slotting through conformal mapping. Neither of the above methods can accurately describe the geometry of stator/rotor slots and their slotting effect. Innovatively, the SD techniques can accurately describe complicated geometric structures and calculate the influence of stator/rotor slots on the air-gap magnetic field^17,18^. However, an essential premise of the SD technique is to assume infinitely permeable iron, ignoring its nonlinearity (i.e., *B*-*H* magnetization curve). Therefore, the magnetic saturation effect cannot be considered, resulting in overestimating flux density and electromagnetic torque^19,20^.

Some hybrid field models have recently been proposed to account for saturation by combining the EMC, CT, SD, and complex permeance model (CPM). Wu et al.^21^. proposed a nonlinear hybrid field model, which has the synergies of both MEC and CPM. The saturation effect is accounted for by considering the potential magnetic distribution on the stator bore. Hanic et al.^22^. presented a hybrid CT and MEC model. The saturation effect is considered by using additional point wires in the inter-polar and slots region. The combined model of the LMCM and SD techniques was also used to analyze spoke-type PM in-wheel machines^23^. The LMCM considers the magnetic saturation of stator teeth. However, the above hybrid approach cannot accurately characterize the complex geometric structures compared with the SD techniques.

Sprangers et al.^24^. proposed a multi-layer analytical model based on the complex Fourier method. The permeability variation of the slotted structure is embedded directly in the magnetic field solution with the convolution theorem, and the iron is no longer assumed to have infinite permeability. The semi-analytical method considers the finite iron permeability in many PM synchronous machines, including surface-mounted, surface-embedded, and spoke-type topologies^25^. Given that the method can consider the finite iron permeability, Liang and Djelloul-Khedda et al.^26,27^. combined the transient Bertotti’s model to predict the iron-core losses of a spoke-type PM in-wheel machine for EVs and flux-modulated PM synchronous machines. In addition, combining multi-layer models and corresponding iterative algorithms can explain the nonlinear characteristics of iron permeability. Djelloul-Khedda et al.^28^. applied it to the nonlinear analytical prediction of magnetic field and electromagnetic performance for switched reluctance machines. However, the permeability of the stator- and rotor yoke is still infinite. Zhang et al.^29^. proposed a magnetic field analytical model of the segmented Halbach array machines, considering the nonlinear properties of all irons. Taking each tooth-tip as a single region cannot describe the uneven flux density distribution. Note that the tooth-tips are more prone to saturation, accompanied by non-uniform flux density distribution.

When the machine runs under load, there is magnetic saturation, especially in the stator tooth-tips, which is more significant, accompanied by the uneven permeability distribution. Based on the literature^4^, this paper proposes a magnetic field analytical model of surface-embedded PM in-wheel machines considering iron nonlinearity. In addition, we subdivided the stator tooth-tip into three sub-regions in the circumferential direction to overcome the problem of significant uneven distribution of magnetic permeability in the stator tooth-tips. The main innovation and contribution is that the field model considers all iron’s permeability nonlinearity. In addition, the tooth-tip is subdivided into three sub-regions to describe the uneven flux density distribution accurately. Solving the field matrix equation using the corresponding boundary conditions, the nonlinear analytical model obtains the electromagnetic performance, namely, the magnetic flux density, back-EMF, torque, etc. Subsequently, the FEA and prototype bench experiment verify the proposed model’s accuracy.

**Fig. 1:**
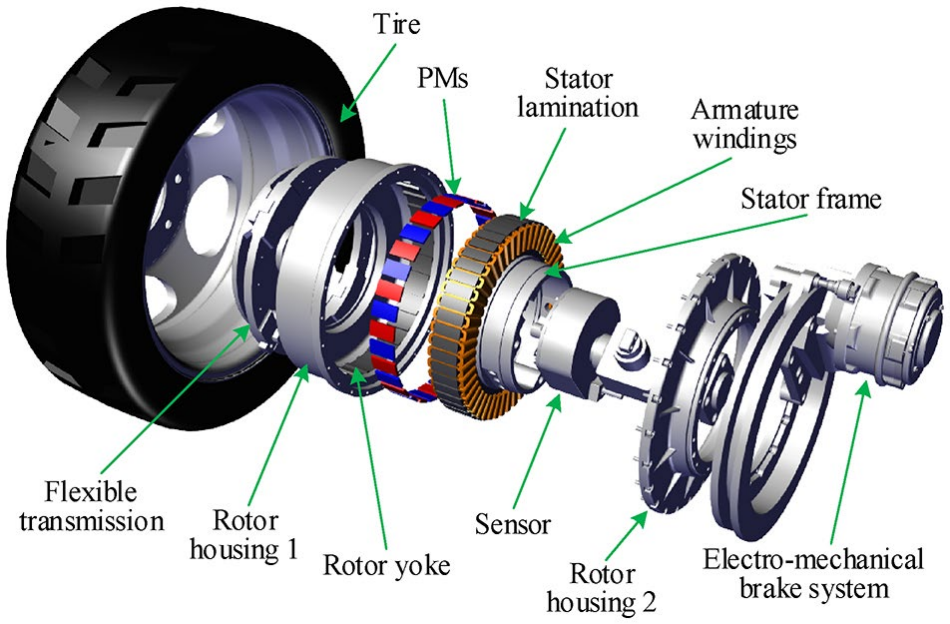
Electric vehicle wheel assembly driven by in-wheel machine^4^.

## Analytical model of in-wheel machine

**Fig. 2:**
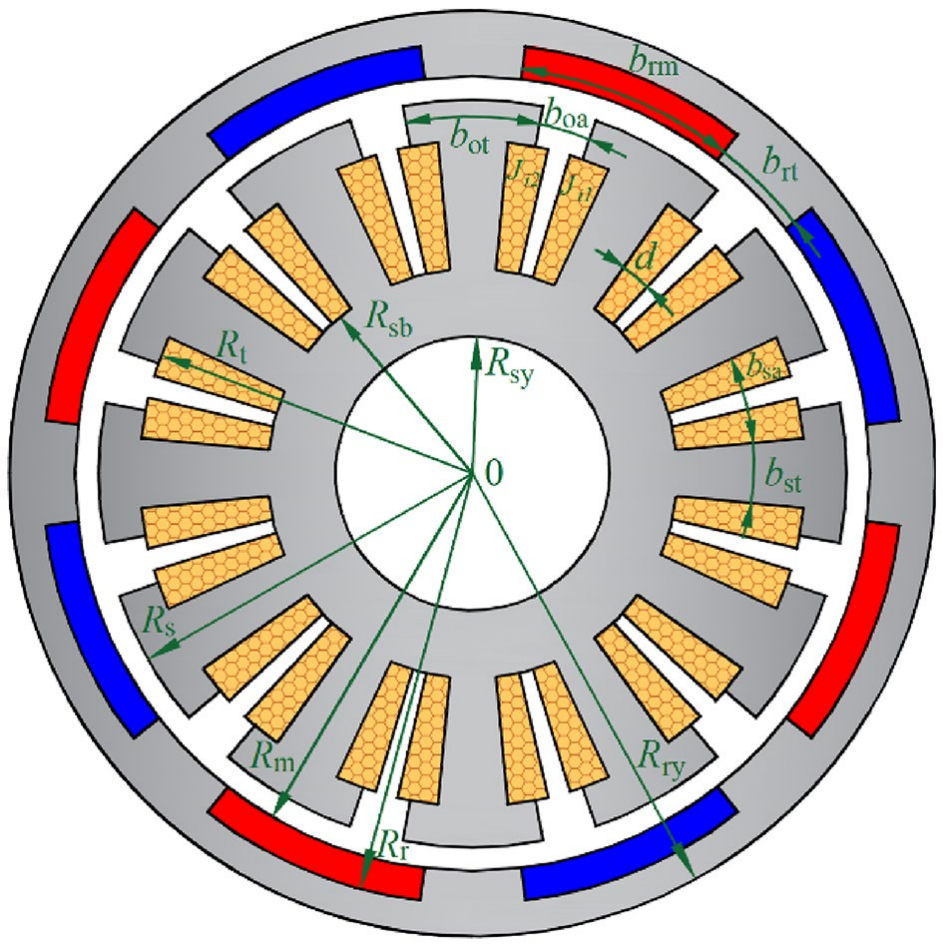
The cross-section diagram of the in-wheel machine.

Figure 1 shows the electric wheel assembly driven by an in-wheel machine, which significantly improves the efficiency of the transmission system. Figure 2 shows a two-dimensional schematic diagram of the in-wheel machine. In the polar coordinate system, the in-wheel machine is divided into six subdomains in the radial direction, including stator yoke (Region I), stator slot and stator tooth (Region II), tooth tip and slot opening (Region III), air-gap (Region IV), rotor tooth and rotor slot (Region V), and rotor yoke (Region VI). The inner diameter of the stator yoke, the inner diameter of the stator slot, the outer diameter of the stator tooth, the inner diameter of the rotor, the outer diameter of the rotor slot, and the outer diameter of the rotor yoke are marked as *R*_sy_, *R*_sb_, *R*_s_, *R*_m_, *R*_r_, and *R*_ry_, respectively. The opening angle of slot-openings, tooth-tips, stator slots, stator teeth, rotor slots, rotor teeth, and armature windings are marked as *b*_oa_, *b*_ot_, *b*_sa_, *b*_st_, *b*_rm_, *b*_rt_, and *d*, respectively. In order to simplify the analytical model, the eddy current effect and end effect of the motor are neglected.

###  Excitation source

For simplification, the complex expression of the general Fourier series is defined as follows:

1 $$\Lambda \left( {r,\theta } \right)=\Re e\left\{ {\sum\limits_{{n= - N}}^{N} {{{\hat {\Lambda }}_n}\left( r \right){e^{ - jn\theta }}} } \right\}$$

2 $$\Gamma \left( \theta \right)=\Re e\left\{ {\sum\limits_{{n= - N}}^{N} {{{\hat {\Gamma }}_n}{e^{ - jn\theta }}} } \right\}$$

where, Λ can represent MVP (*A*_z_) and magnetic flux density (*B*_*r*_, *B*_*θ*_). Γ can, respectively, represent current density (*J*_z_), magnetization (*M*_*r*_, *M*_*θ*_), and relative permeability (*µ*), and *N* denotes the maximum spatial harmonic order.

The arrangement of the double-layer windings is shown in Fig. 2. According to the periodicity, the coefficients of the complex Fourier series of the current density of the armature windings are expressed as:

3 $${\hat {J}_{\text{z},n}}=\frac{1}{{2\pi jn}}\sum\limits_{{i=1}}^{{{Q_{\text{s}}}}} {{J_{i,1}}{e^{ - jn\frac{{{b_{{\text{sa}}}}}}{2}}}\left[ {\left( {{e^{jnd}} - 1} \right)+{J_{i,2}}{e^{jn\frac{{{b_{{\text{sa}}}}}}{2}}}\left( {1 - {e^{ - jnd}}} \right)} \right]{e^{jn{\theta _i}}}}$$

*θ*_*i*_ is the angle of the center of the *i*-th stator slot, and *Q*_s_ is the number of the stator slots.

**Fig. 3:**
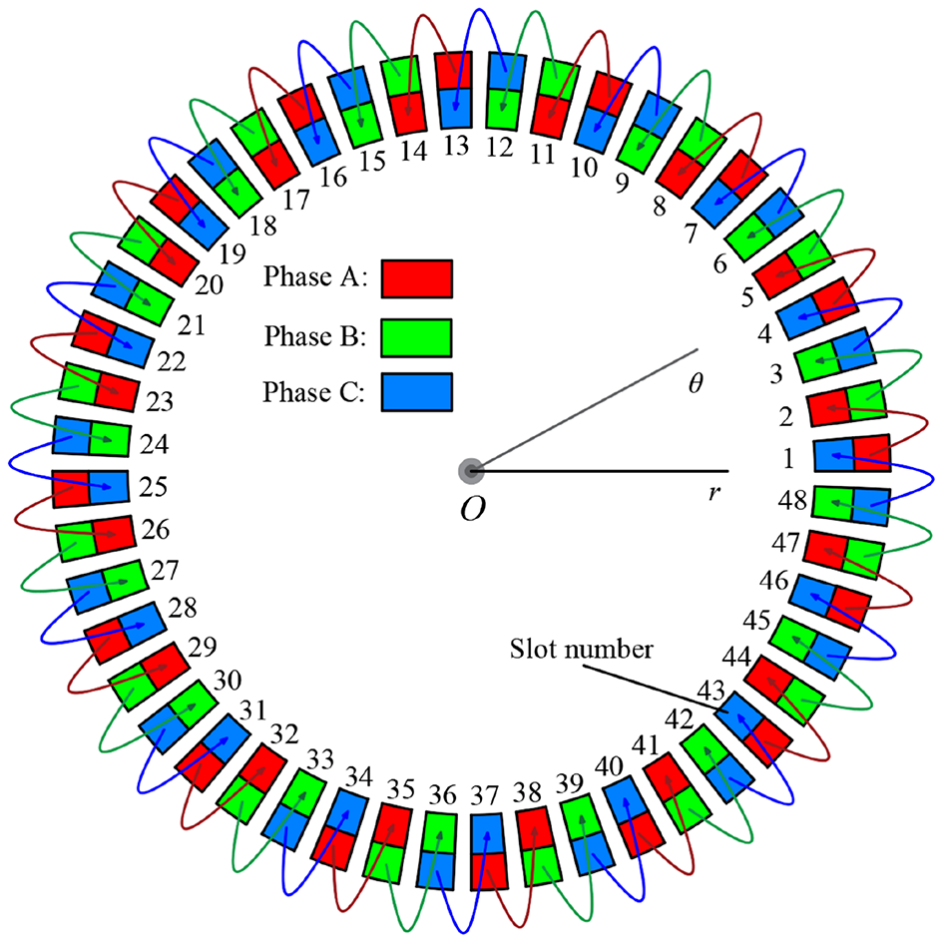
Stator armature winding layout.

**Fig. 4:**
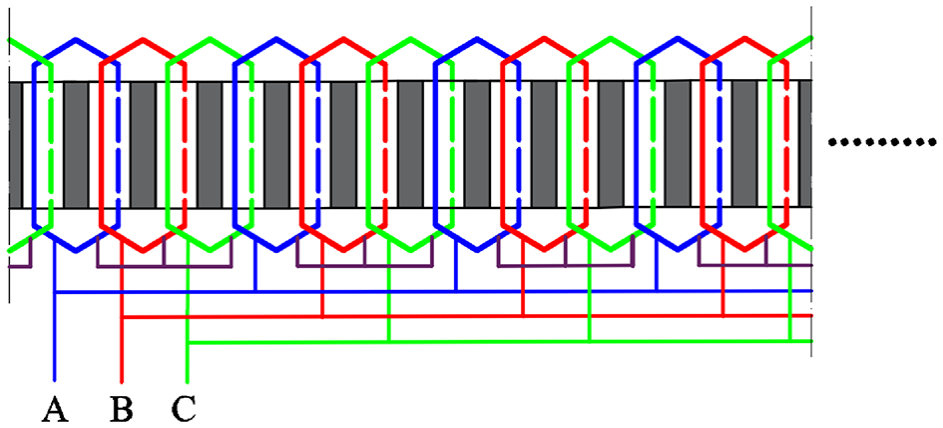
Winding expansion diagram.

The connection form of the three-phase armature winding is shown in Fig. 3, which is a double-layer overlapping winding topology. Figure 4 shows the expansion diagram of the armature windings. Therefore, the current density of the double-layer armature winding can be calculated using the following formulas:

4 $${J_{i,{\text{1}}}}=\frac{{{N_{\text{c}}}}}{{{A_{\text{c}}}}}C_{1}^{{\text{T}}}\left[ {{i_{\text{A}}}\;\;\;{i_{\text{B}}}\;\;\;{i_{\text{C}}}} \right]$$

5 $${J_{i,2}}=\frac{{{N_{\text{c}}}}}{{{A_{\text{c}}}}}C_{2}^{{\text{T}}}\left[ {{i_{\text{A}}}\;\;\;{i_{\text{B}}}\;\;\;{i_{\text{C}}}} \right]$$

6 $${A_{\text{c}}}=d\left( {R_{{\text{t}}}^{2} - R_{{{\text{sb}}}}^{2}} \right)/2$$

where, *N*_c_ is the number of coils per slot, and *A*_c_ is the one coil side area, Eqs. (7)−(10) give the matrix corresponding to the fractional-slot concentrated winding adopted by the in-wheel machines.

7 $${C_1}={\left[ {{\vartheta _{\text{a}}},\;\;{\vartheta _{\text{a}}},\;\; \cdots ,\;\;{\vartheta _{\text{a}}},\;\;{\vartheta _{\text{a}}}} \right]_{1 \times 8}}$$

8 $${\vartheta _1}=\left[ {\begin{array}{*{20}{c}} 0&{ - 1}&0&0&{ - 1}&0 \\ 0&0&{ - 1}&0&0&{ - 1} \\ { - 1}&0&0&{ - 1}&0&0 \end{array}} \right]$$

9 $${C_2}={\left[ {{\vartheta _{\text{b}}},\;\;{\vartheta _{\text{b}}},\;\; \cdots ,\;\;{\vartheta _{\text{b}}},\;\;{\vartheta _{\text{b}}}} \right]_{1 \times 8}}$$

10 $${\vartheta _2}=\left[ {\begin{array}{*{20}{c}} 1&0&0&1&0&0 \\ 0&1&0&0&1&0 \\ 0&0&1&0&0&1 \end{array}} \right]$$

**Fig. 5:**
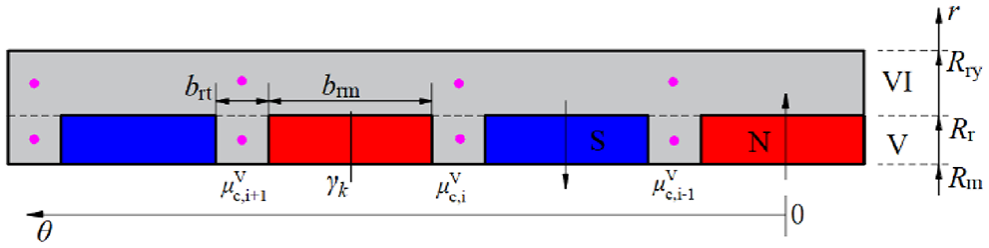
Expansion diagram of the outer rotor structure with surface-embedded PMs.

The schematic diagram of the outer rotor unfolding in the circumferential direction is shown in Fig. 5. According to the periodicity of the magnet arrangement, expanding *M*_*r*,*n*,_ and *M*_*θ*,*n*_ into complex Fourier series over the circumference is as follows:

11 $$M\left( \theta \right)=\sum\limits_{{n= - N}}^{N} {{{\hat {M}}_n}{e^{ - jn\theta }}}$$

**Fig. 6:**
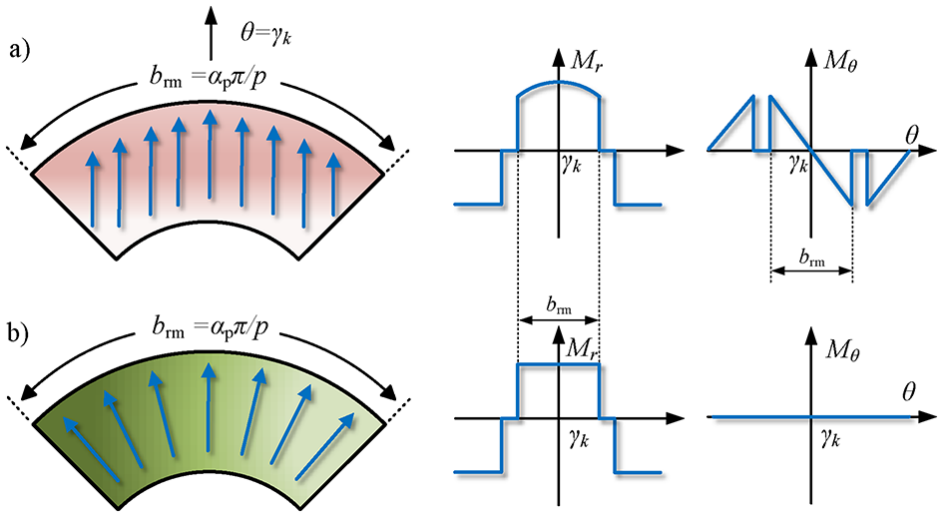
Magnetization patterns of the PMs. (**a**) Parallel magnetization. (**b**) Radial magnetization.

As shown in Fig. 6, magnets are usually magnetized in radial and tangential directions. For radial magnetization, the Fourier series coefficients (${\hat {M}_{r,n}}$, ${\hat {M}_{\theta ,n}}$) can be calculated respectively via

12 $${\hat {M}_{r,n}}=\frac{{{B_{{\text{rem}}}}}}{{2\pi {\mu _0}}}\sum\limits_{{k=1}}^{p} {\left\{ {\int_{{{\gamma _k} - {b_{{\text{rt}}}} - \frac{{3{b_{{\text{rm}}}}}}{2}}}^{{{\gamma _k} - {b_{{\text{rt}}}} - \frac{{{b_{{\text{rm}}}}}}{2}}} { - {e^{jn\theta }}d\theta } +\int_{{{\gamma _k} - \frac{{{b_{{\text{rm}}}}}}{2}}}^{{{\gamma _k}+\frac{{{b_{{\text{rm}}}}}}{2}}} {{e^{jn\theta }}d\theta } } \right\}}$$

13 $${\hat {M}_{\theta ,n}}=0$$

Similarly, for parallel magnetization,

14 $$\begin{gathered} {{\hat {M}}_{r,n}}= - \frac{{{B_{{\text{rem}}}}}}{{2\pi {\mu _0}}}\sum\limits_{{k=1}}^{p} {\left\{ {\int_{{{\gamma _k}+{b_{{\text{rt}}}}+\frac{{{b_{{\text{rm}}}}}}{2}}}^{{{\gamma _k}+{b_{{\text{rt}}}}+{b_{{\text{rm}}}}}} {\cos \left[ {\theta - \left( {{\gamma _k}+{b_{{\text{rt}}}}+{b_{{\text{rm}}}}} \right)} \right]{e^{jn\theta }}d\theta } } \right.} \hfill \\ {\text{ }} - \int_{{{\gamma _k} - \frac{{{b_{{\text{rm}}}}}}{2}}}^{{{\gamma _k}+\frac{{{b_{{\text{rm}}}}}}{2}}} {\cos \left( {\theta - {\gamma _k}} \right){e^{jn\theta }}d\theta } \hfill \\ {\text{ }}\left. {{\text{ }}+\int_{{{\gamma _k} - {b_{{\text{rt}}}} - {b_{{\text{rm}}}}}}^{{{\gamma _k} - {b_{{\text{rt}}}} - \frac{{{b_{{\text{rm}}}}}}{2}}} {\cos \left[ {\theta - \left( {{\gamma _k} - {b_{{\text{rt}}}} - {b_{{\text{rm}}}}} \right)} \right]{e^{jn\theta }}d\theta } } \right\} \hfill \\ \end{gathered}$$

15 $$\begin{gathered} {{\hat {M}}_{\theta ,n}}= - \frac{{{B_{{\text{rem}}}}}}{{2\pi {\mu _0}}}\sum\limits_{{k=1}}^{p} {\left\{ {\int_{{{\gamma _k}+{b_{{\text{rt}}}}+\frac{{{b_{{\text{rm}}}}}}{2}}}^{{{\gamma _k}+{b_{{\text{rt}}}}+{b_{{\text{rm}}}}}} {\sin \left[ {\theta - \left( {{\gamma _k}+{b_{{\text{rt}}}}+{b_{{\text{rm}}}}} \right)} \right]{e^{jn\theta }}d\theta } } \right.} \hfill \\ {\text{ }} - \int_{{{\gamma _k} - \frac{{{b_{{\text{rm}}}}}}{2}}}^{{{\gamma _k}+\frac{{{b_{{\text{rm}}}}}}{2}}} {\sin \left( {\theta - {\gamma _k}} \right){e^{jn\theta }}d\theta } \hfill \\ {\text{ }}\left. {{\text{ }}+\int_{{{\gamma _k} - {b_{{\text{rt}}}} - {b_{{\text{rm}}}}}}^{{{\gamma _k} - {b_{{\text{rt}}}} - \frac{{{b_{{\text{rm}}}}}}{2}}} {\sin \left[ {\theta - \left( {{\gamma _k} - {b_{{\text{rt}}}} - {b_{{\text{rm}}}}} \right)} \right]{e^{jn\theta }}d\theta } } \right\} \hfill \\ \end{gathered}$$

where, *B*_rem_ represents the magnets’ remanence, and *p* is the pole pair numbers of the magnets, the permeability of vacuum *µ*_0_ = 4π × 10^− 7^ H/m.

**Fig. 7:**
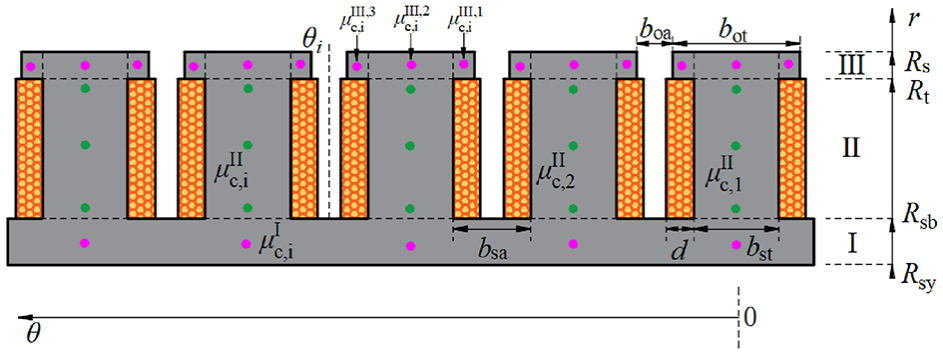
Expansion diagram of the stator in the polar coordinate system.

### Permeability distribution

Figure 7 shows the stator’s expansion diagram and gives the sub-regions division principle. For the stator, the tooth-tips are prone to magnetic saturation under on-load conditions. In particular, to accurately describe the inhomogeneity of the flux density distribution in the tooth-tips, we subdivided each tooth-tip into three regions along the circumferential direction. That is, the relative permeability in the *i*-th tooth-tip is represented by $\:{\text{μ}}_{\text{c,\:}\text{i}}^{\text{III}\text{,1}}$, $\:{\text{μ}}_{\text{c,\:}\text{i}}^{\text{III}\text{,2}}$, and $\:{\text{μ}}_{\text{c,\:}\text{i}}^{\text{III}\text{,3}}$, respectively. According to the periodicity, the relative permeability distribution in Region II and Region III are given by Eqs. (16) and (17), respectively. Similarly, the relative permeability distribution is expressed as Eq. (18) for the rotor topology with surface-embedded magnets shown in Fig. 4.

16 $${\mu ^{{\text{II}}}}(\theta )=\left\{ {\begin{array}{*{20}{l}} {{\mu _0}\;\;\;\;\;\;\;\theta \in \left[ {{\theta _i} - \frac{{{b_{{\text{sa}}}}}}{2},{\theta _i}+\frac{{{b_{{\text{sa}}}}}}{2}} \right]} \\ {\mu _{{{\text{c}},i}}^{{{\text{II}}}}\;\;\;\theta \in \left[ {{\theta _i} - \frac{{{b_{{\text{sa}}}}}}{2} - {b_{{\text{st}}}},{\theta _i} - \frac{{{b_{{\text{sa}}}}}}{2}} \right]} \end{array}} \right.$$

17 $${\mu ^{{\text{III}}}}(\theta )=\left\{ \begin{gathered} \begin{array}{*{20}{l}} {{\mu _0}\;\;\;\;\;\;\;\theta \in \left[ {{\theta _i} - \frac{{{b_{{\text{oa}}}}}}{2},{\theta _i}+\frac{{{b_{{\text{oa}}}}}}{2}} \right]} \\ {\mu _{{{\text{c}},i}}^{{{\text{III}},{\text{1}}}}\;\;\;\theta \in \left[ {{\theta _i} - \frac{1}{2}\left( {{b_{{\text{oa}}}}+{b_{{\text{ot}}}} - {b_{{\text{st}}}}} \right),{\theta _i} - \frac{{{b_{{\text{oa}}}}}}{2}} \right]} \end{array} \hfill \\ \mu _{{{\text{c}},i}}^{{{\text{III,}}2}}\;\;\;\theta \in \left[ {{\theta _i} - \frac{1}{2}\left( {{b_{{\text{oa}}}}+{b_{{\text{ot}}}} - {b_{{\text{st}}}}} \right) - {b_{{\text{st}}}},{\theta _i} - \frac{1}{2}\left( {{b_{{\text{oa}}}}+{b_{{\text{ot}}}} - {b_{{\text{st}}}}} \right)} \right] \hfill \\ \mu _{{{\text{c}},i}}^{{{\text{III,3}}}}\;\;\;\theta \in \left[ {{\theta _i} - \frac{{{b_{{\text{oa}}}}}}{2} - {b_{{\text{ot}}}},{\theta _i} - \frac{{{b_{{\text{oa}}}}}}{2} - \frac{1}{2}\left( {{b_{{\text{ot}}}} - {b_{{\text{st}}}}} \right)} \right] \hfill \\ \end{gathered} \right.$$

18 $${\mu ^{\text{V}}}(\theta )=\left\{ {\begin{array}{*{20}{l}} {{\mu _0}{\mu _{\text{r}}}\;\;\;\;\;\;\;\theta \in \left[ {{\theta _i} - \frac{{{b_{{\text{rm}}}}}}{2},{\theta _i}+\frac{{{b_{{\text{rm}}}}}}{2}} \right]} \\ {\mu _{{{\text{c}},i}}^{{\text{V}}}\;\;\;\theta \in \left[ {{\theta _i} - \frac{{{b_{{\text{rm}}}}}}{2} - {b_{{\text{rt}}}},{\theta _i} - \frac{{{b_{{\text{rm}}}}}}{2}} \right]} \end{array}} \right.$$

where $\:{\text{μ}}_{\text{c,}\text{\:}\text{i}}^{\text{II}}$, $\:{\text{μ}}_{\text{c,}\text{\:}\text{i}}^{\text{III}}$, and $\:{\text{μ}}_{\text{c}\text{,}\text{\:}\text{i}}^{\text{V}}$ are, respectively, the relative permeability of the silicon steel sheet in Regions II, III, and V. The complex Fourier coefficients are then derived from the periodicity of the permeability distribution as follows:

19 $$\hat {\mu }_{n}^{{{\text{II}}}}=\left\{ {\begin{array}{*{20}{l}} \begin{gathered} \frac{1}{{2\pi jn}}\sum\limits_{{i=1}}^{{{Q_{\text{s}}}}} {\left[ {\mu _{{{\text{c}},i}}^{{{\text{II}}}}{e^{ - jn\frac{{{b_{{\text{sa}}}}}}{2}}}\left( {1 - {e^{ - jn{b_{{\text{st}}}}}}} \right)} \right.} \hfill \\ \left. {{\text{ }}+2j{\mu _0}\sin \left( {\frac{{n{b_{{\text{sa}}}}}}{2}} \right)} \right]{e^{jn{\theta _i}}}{\text{ }}n \ne {\text{0}} \hfill \\ \end{gathered} \\ {\frac{1}{{2\pi }}\sum\limits_{{i=1}}^{{{Q_{\text{s}}}}} {\left( {{\mu _0}{b_{{\text{sa}}}}+\mu _{{{\text{c}},i}}^{{{\text{II}}}}{b_{{\text{st}}}}} \right)} {\text{ }}n{\text{=0}}} \end{array}} \right.$$

20 $$\hat {\mu }_{n}^{{{\text{III}}}}=\left\{ {\begin{array}{*{20}{l}} \begin{gathered} \frac{1}{{2\pi jn}}\sum\limits_{{i=1}}^{{{Q_{\text{s}}}}} {\left[ \begin{gathered} \frac{{{\mu _0}}}{{jn}}{e^{jn{\theta _i}}}2j\sin \left( {\frac{{n{b_{{\text{oa}}}}}}{2}} \right) \hfill \\ +\frac{{\mu _{{{\text{c}},i}}^{{{\text{III,1}}}}}}{{jn}}{e^{jn\left( {{\theta _i} - \frac{{{b_{{\text{oa}}}}}}{2}} \right)}}\left( {1 - {e^{ - jn\frac{1}{2}\left( {{b_{{\text{ot}}}} - {{\text{b}}_{{\text{st}}}}} \right)}}} \right) \hfill \\ +\frac{{\mu _{{{\text{c}},i}}^{{{\text{III,2}}}}}}{{jn}}{e^{jn\left( {{\theta _i} - \frac{{{b_{{\text{oa}}}}}}{2} - \frac{1}{2}\left( {{b_{{\text{ot}}}} - {{\text{b}}_{{\text{st}}}}} \right)} \right)}}\left( {1 - {e^{ - jn{{\text{b}}_{{\text{st}}}}}}} \right) \hfill \\ +\frac{{\mu _{{{\text{c}},i}}^{{{\text{III,3}}}}}}{{jn}}{e^{jn\left( {{\theta _i} - \frac{1}{2}\left( {{b_{{\text{oa}}}}+{b_{{\text{ot}}}}{\text{-}}{{\text{b}}_{{\text{st}}}}} \right)} \right)}}\left( {1 - {e^{ - jn\frac{1}{2}\left( {{b_{{\text{ot}}}} - {{\text{b}}_{{\text{st}}}}} \right)}}} \right) \hfill \\ \end{gathered} \right]} {\text{ }}n \ne {\text{0}} \hfill \\ \hfill \\ \end{gathered} \\ {\frac{1}{{2\pi }}\sum\limits_{{i=1}}^{{{Q_{\text{s}}}}} {\left[ \begin{gathered} {\mu _0}{b_{{\text{oa}}}}+\frac{1}{2}\mu _{{{\text{c}},i}}^{{{\text{III,1}}}}\left( {{b_{{\text{ot}}}} - {{\text{b}}_{{\text{st}}}}} \right) \hfill \\ +\mu _{{{\text{c}},i}}^{{{\text{III,2}}}}{b_{{\text{st}}}}+\frac{1}{2}\mu _{{{\text{c}},i}}^{{{\text{III,3}}}}\left( {{b_{{\text{ot}}}} - {{\text{b}}_{{\text{st}}}}} \right) \hfill \\ \end{gathered} \right]} {\text{ }}n{\text{=0}}} \end{array}} \right.$$

21 $$\hat {\mu }_{n}^{{\text{V}}}=\left\{ {\begin{array}{*{20}{l}} \begin{gathered} \frac{1}{{2\pi jn}}\sum\limits_{{i=1}}^{{2P}} {\left[ {\mu _{{{\text{c}},i}}^{{\text{V}}}{e^{ - jn\frac{{{b_{{\text{rm}}}}}}{2}}}\left( {1 - {e^{ - jn{b_{{\text{rt}}}}}}} \right)} \right.} \hfill \\ {\text{ }}+2j{\mu _0}\sin \left( {\frac{{n{b_{{\text{rm}}}}}}{2}} \right){e^{jn{\theta _i}}}{\text{ }}n \ne 0 \hfill \\ \end{gathered} \\ {\frac{1}{{2\pi }}\sum\limits_{{i=1}}^{{{Q_{\text{s}}}}} {\left( {{\mu _0}{\mu _{\text{r}}}{b_{{\text{rm}}}}+\mu _{{{\text{c}},i}}^{{\text{V}}}{b_{{\text{rt}}}}} \right)} {\text{ }}n{\text{=0}}} \end{array}} \right.$$

The slotted topology of the stator and rotor has different relative permeability in different media, making the permeability discontinuous in the tangential direction. Therefore, the fast Fourier decomposition method is used to solve the tangential component of the coefficient. That is, $\:\left\{{\text{μ}}_{\text{0}}\text{,\:}{\text{μ}}_{\text{c,\:}\text{i}}^{\text{II}}\text{,\:}{\text{μ}}_{\text{c,\:}\text{i}}^{\text{III}\text{,1}}\text{,\:}{\text{μ}}_{\text{c,\:}\text{i}}^{\text{III}\text{,2}}\text{,}{\text{\:}\text{μ}}_{\text{c,\:}\text{i}}^{\text{III}\text{,3}}\text{,\:}{\text{μ}}_{\text{c,\:}\text{i}}^{\text{V}}\right\}$ in Eqs. (19)−(21) are replaced by $\:\left\{1/{\text{μ}}_{\text{0}}\text{,\:}\text{1}/{\text{μ}}_{\text{c,\:}\text{i}}^{\text{II}}\text{,\:}\text{1}/{\text{μ}}_{\text{c,\:}\text{i}}^{\text{III}\text{,1}}\text{,}\text{1}/{\text{μ}}_{\text{c,\:}\text{i}}^{\text{III}\text{,2}}\text{,}\text{1}/{\text{μ}}_{\text{c,\:}\text{i}}^{\text{III}\text{,3}}\text{,\:}\text{1}/{\text{μ}}_{\text{c,\:}\text{i}}^{\text{V}}\right\}$, respectively^25,28^.

### General solutions for magnetic field

Regarding the MVP, there is no excitation source in the region of air and iron, which satisfies Laplace’s equation (i.e., Regions I, III, IV, and VI). Conversely, the stator and rotor slots have excitation sources (viz., armature windings and magnets), so the MVP satisfies Poisson’s equation (i.e., Regions II and V). The governing equations for each region are represented as second-order differential equations as follows:

22 $$\begin{gathered} \frac{{{{\left. {{\partial ^2}{\mathbf{A}}_{\text{z}}^{i}} \right|}_r}}}{{\partial {r^2}}}+\frac{1}{r}\frac{{{{\left. {\partial {\mathbf{A}}_{\text{z}}^{i}} \right|}_r}}}{{\partial r}} - {\left( {\frac{{{{\mathbf{V}}^i}}}{r}} \right)^2}{\left. {{\mathbf{A}}_{\text{z}}^{i}} \right|_r} \hfill \\ \;=\left\{ {\begin{array}{*{20}{l}} {0,\;\;\;\;\;\;\;\;\;\;\;\;\;\;\;\;\;\;\;\;\;\;\;\;{\text{ }}\;{\text{ }}i={\text{I}},{\text{ III, IV, VI,}}} \\ \begin{gathered} - {\mathbf{\mu }}_{{{\text{c}},\theta }}^{i}{{\mathbf{J}}_z},\;\;\;\;\;\;\;\;\;\;\;\;\;\;\;\;\;{\text{ }}i={\text{II}}, \hfill \\ - \frac{{{\mu _0}}}{r}\left( {{{\mathbf{M}}_\theta }+j{\mathbf{\mu }}_{{{\text{c,}}\theta }}^{{\text{V}}}{\mathbf{N}}{{\left[ {{\mathbf{\mu }}_{{{\text{c, }}r}}^{{\text{V}}}} \right]}^{ - 1}}{{\mathbf{M}}_r}} \right),\;\;\;{\text{ }}\;i={\text{V}}{\text{.}} \hfill \\ \end{gathered} \end{array}} \right. \hfill \\ \end{gathered}$$

where

23 $${\mathbf{N}}={\text{diag}}\left( { - N,{\text{ }} \ldots ,{\text{ }} - 1,{\text{ }}1,{\text{ }} \cdots ,{\text{ }}N} \right)$$

24 $${{\mathbf{V}}^i}=\left\{ {\begin{array}{*{20}{l}} {{\mathbf{N}},{\text{ }}i{\text{ = I, IV, and VI}}} \\ {{\mathbf{\mu }}_{{{\text{c}},\theta }}^{i}{\mathbf{N}}{{\left[ {{\mathbf{\mu }}_{{{\text{c}},r}}^{i}} \right]}^{ - 1}}{\mathbf{N}},{\text{ }}i{\text{ = II, III, and V}}} \end{array}} \right.$$

25 $${{\mathbf{M}}_r}={\left[ {{{\hat {M}}_{r, - N}}{\text{, }} \ldots ,{\text{ }}{{\hat {M}}_{r, - 1}}{\text{, }}{{\hat {M}}_{r,1}}{\text{, }} \ldots ,{\text{ }}{{\hat {M}}_{r,N}}} \right]^{\text{T}}}$$

26 $${{\mathbf{M}}_\theta }={\left[ {{{\hat {M}}_{\theta , - N}}{\text{, }} \ldots ,{\text{ }}{{\hat {M}}_{\theta , - 1}}{\text{, }}{{\hat {M}}_{\theta ,1}}{\text{, }} \ldots ,{\text{ }}{{\hat {M}}_{\theta ,N}}} \right]^{\text{T}}}$$

27 $${{\mathbf{J}}_\text{z}}={\left[ {{{\mathbf{J}}_\text{z}}_{{, - N}}{\text{, }} \ldots ,{\text{ }}{{\mathbf{J}}_\text{z}}_{{, - 1}}{\text{, }}{{\mathbf{J}}_\text{z}}_{{,1}}{\text{, }} \ldots ,{\text{ }}{{\mathbf{J}}_\text{z}}_{{,N}}} \right]^{\text{T}}}$$

By solving governing differential Eq. (22), we can get the general solution expression of MVP in each sub-region, as follows:

28 $${\mathbf{A}}_{\mathcal{z}}^{{\text{I}}}=\left[ {{{\mathbf{\xi }}^{\text{I}}}{{\left( {\frac{r}{{{R_{{\text{sb}}}}}}} \right)}^{{{\mathbf{\lambda }}^I}}}{{\mathbf{\alpha }}^{\text{I}}}+{{\mathbf{\xi }}^{\text{I}}}{{\left( {\frac{{{R_{{\text{sy}}}}}}{r}} \right)}^{{{\mathbf{\lambda }}^{\text{I}}}}}{{\mathbf{\beta }}^{\text{I}}}} \right]{e^{ - j{\mathbf{N}}\theta }}$$

29 $${\mathbf{A}}_{\mathcal{z}}^{{{\text{II}}}}=\left[ {{{\mathbf{\xi }}^{{\text{II}}}}{{\left( {\frac{r}{{{R_{\text{t}}}}}} \right)}^{{{\mathbf{\lambda }}^{{\text{II}}}}}}{{\mathbf{\alpha }}^{{\text{II}}}}+{{\mathbf{\xi }}^{{\text{II}}}}{{\left( {\frac{{{R_{{\text{sb}}}}}}{r}} \right)}^{{{\mathbf{\lambda }}^{{\text{II}}}}}}{{\mathbf{\beta }}^{{\text{II}}}}+{r^2}{{\mathbf{F}}^{{\text{II}}}}} \right]{e^{ - j{\mathbf{N}}\theta }}$$

30 $${\mathbf{A}}_{\mathcal{z}}^{{{\text{III}}}}=\left[ {{{\mathbf{\xi }}^{{\text{III}}}}{{\left( {\frac{r}{{{R_{\text{s}}}}}} \right)}^{{{\mathbf{\lambda }}^{I{\text{I}}I}}}}{{\mathbf{\alpha }}^{{\text{III}}}}+{{\mathbf{\xi }}^{{\text{III}}}}{{\left( {\frac{{{R_{\text{t}}}}}{r}} \right)}^{{{\mathbf{\lambda }}^{{\text{III}}}}}}{{\mathbf{\beta }}^{{\text{III}}}}} \right]{e^{ - j{\mathbf{N}}\theta }}$$

31 $${\mathbf{A}}_{\text{z}}^{{{\text{IV}}}}=\left[ {{{\left( {\frac{r}{{{R_{\text{m}}}}}} \right)}^{{{\mathbf{\lambda }}^{{\text{IV}}}}}}{{\mathbf{\alpha }}^{{\text{IV}}}}+{{\left( {\frac{{{R_{\text{s}}}}}{r}} \right)}^{{{\mathbf{\lambda }}^{{\text{IV}}}}}}{{\mathbf{\beta }}^{{\text{IV}}}}} \right]{e^{ - j{\mathbf{N}}\theta }}$$

32 $${\mathbf{A}}_{\mathcal{z}}^{{\text{V}}}=\left[ {{{\mathbf{\xi }}^{\text{V}}}{{\left( {\frac{r}{{{R_{\text{r}}}}}} \right)}^{{{\mathbf{\lambda }}^{\text{V}}}}}{{\mathbf{\alpha }}^{\text{V}}}+{{\mathbf{\xi }}^{\text{V}}}{{\left( {\frac{{{R_{\text{m}}}}}{r}} \right)}^{{{\mathbf{\lambda }}^{\text{V}}}}}{{\mathbf{\beta }}^{\text{V}}}+r{{\mathbf{F}}^{\text{V}}}} \right]{e^{ - j{\mathbf{N}}\theta }}$$

33 $${\mathbf{A}}_{\mathcal{z}}^{{{\text{VI}}}}=\left[ {{{\mathbf{\xi }}^{{\text{VI}}}}{{\left( {\frac{r}{{{R_{{\text{ry}}}}}}} \right)}^{{{\mathbf{\lambda }}^{{\text{VI}}}}}}{{\mathbf{\alpha }}^{{\text{VI}}}}+{{\mathbf{\xi }}^{{\text{VI}}}}{{\left( {\frac{{{R_{\text{r}}}}}{r}} \right)}^{{{\mathbf{\lambda }}^{{\text{VI}}}}}}{{\mathbf{\beta }}^{{\text{VI}}}}} \right]{e^{ - j{\mathbf{N}}\theta }}$$

where,

34 $${{\mathbf{F}}^{{\text{II}}}}={\left( {{{\mathbf{V}}^{{\text{II}}}} - 4{\mathbf{I}}} \right)^{ - 1}}{\mathbf{\mu }}_{{{\text{c}},\theta }}^{{{\text{II}}}}{{\mathbf{J}}_\text{z}}$$

35 $${{\mathbf{F}}^{\text{V}}}={\mu _0}{\left( {{{\mathbf{V}}^{\text{V}}} - {\mathbf{I}}} \right)^{ - 1}}\left( {{{\mathbf{M}}_\theta }+j{\mathbf{\mu }}_{{{\text{c,}}\theta }}^{{\text{V}}}{\mathbf{N}}{{\left[ {{\mathbf{\mu }}_{{{\text{c, }}r}}^{{\text{V}}}} \right]}^{ - 1}}{{\mathbf{M}}_r}} \right)$$

36 $${{\varvec{\mu }}_{{\text{c,}}r}} = \left[ {\begin{array}{lll} {{{\hat \mu }_0}} \hfill & \ldots \hfill & {{{\hat \mu }_{ - 2N}}} \hfill \\ {\; \vdots } \hfill & \ddots \hfill & {\; \vdots } \hfill \\ {{{\hat \mu }_{2N}}} \hfill & \ldots \hfill & {{{\hat \mu }_0}} \hfill \\ \end{array}} \right]$$

37 $${{\bf{\mu }}_{{\text{c,}}\theta }} = {[{\bf{\mu }}_{{\text{c,}}\theta }^{{\text{inv}}}]^{ - 1}} = {\left[ {\begin{array}{lll} {\hat \mu _{\text{0}}^{{\text{inv}}}} \hfill & \ldots \hfill & {\hat \mu _{ - 2N}^{{\text{inv}}}} \hfill \\ {\;\;\; \vdots } \hfill & \ddots \hfill & {\;\;\; \vdots } \hfill \\ {\hat \mu _{2N}^{{\text{inv}}}} \hfill & \ldots \hfill & {\hat \mu _{\text{0}}^{{\text{inv}}}} \hfill \\ \end{array}} \right]^{ - 1}}$$

with **λ**^*i*^ and **ξ**^*i*^ being the diagonal eigenvalue and eigenvector matrix of the **V**^*i*^, respectively. The unknown coefficient matrices **α**^*i*^ and **β**^*i*^ can be obtained with the corresponding boundary conditions.

### Solution for harmonic coefficients

Based on the general solution of the MVP in six subdomains, boundary conditions between adjacent sub-regions can be used to determine the unknown coefficient matrix in the MVP. Given the magnetic field’s continuity, Namely, the normal flux density and the tangential magnetic field strength at the radii $\:{\text{R}}_{\text{sb}}$, $\:{\text{R}}_{\text{t}}$, $\:{\text{R}}_{\text{s}}$, $\:{\text{R}}_{\text{m}}$, and $\:{\text{R}}_{\text{r}}$ of the two adjacent regions are all equal, as shown in Eqs. (38)−(42). Besides, according to the Dirichlet boundary condition, no MVP exists outside the solution domain, as shown in Eq. (43).

Between stator yoke and stator slots/teeth:

38 $${\left. {{\mathbf{A}}_{\text{z}}^{{\text{I}}}} \right|_{r={R_{{\text{sb}}}}}}={\left. {{\mathbf{A}}_{\text{z}}^{{{\text{II}}}}} \right|_{r={R_{{\text{sb}}}}}}, \; \; {\left. {{\mathbf{H}}_{\theta }^{{\text{I}}}} \right|_{r={R_{{\text{sb}}}}}}={\left. {{\mathbf{H}}_{\theta }^{{{\text{II}}}}} \right|_{r={R_{{\text{sb}}}}}}$$

Between stator slots/teeth and slot openings/tooth-tips:

39 $${\left. {{\mathbf{A}}_{\text{z}}^{{{\text{II}}}}} \right|_{r={R_{\text{t}}}}}={\left. {{\mathbf{A}}_{\text{z}}^{{{\text{III}}}}} \right|_{r={R_{\text{t}}}}}, \;\;\; {\left. {{\mathbf{H}}_{\theta }^{{{\text{II}}}}} \right|_{r={R_{\text{t}}}}}={\left. {{\mathbf{H}}_{\theta }^{{{\text{III}}}}} \right|_{r={R_{\text{t}}}}}$$

Between slot-openings/tooth-tips and air-gap:

40 $${\left. {{\mathbf{A}}_{\text{z}}^{{{\text{III}}}}} \right|_{r={R_{\text{s}}}}}={\left. {{\mathbf{A}}_{\text{z}}^{{{\text{IV}}}}} \right|_{r={R_{\text{s}}}}}, \;\; {\left. {{\mathbf{H}}_{\theta }^{{{\text{III}}}}} \right|_{r={R_{\text{s}}}}}={\left. {{\mathbf{H}}_{\theta }^{{{\text{IV}}}}} \right|_{r={R_{\text{s}}}}}$$

Between air-gap and rotor slots/teeth:

41 $${\left. {{\mathbf{A}}_{\text{z}}^{{{\text{IV}}}}} \right|_{r={R_{\text{m}}}}}={\left. {{\mathbf{A}}_{\text{z}}^{{\text{V}}}} \right|_{r={R_{\text{m}}}}}, \;\; {\left. {{\mathbf{H}}_{\theta }^{{{\text{IV}}}}} \right|_{r={R_{\text{m}}}}}={\left. {{\mathbf{H}}_{\theta }^{{\text{V}}}} \right|_{r={R_{\text{m}}}}}$$

Between rotor slots/teeth and rotor yoke:

42 $${\left. {{\mathbf{A}}_{\text{z}}^{{\text{V}}}} \right|_{r={R_{\text{r}}}}}={\left. {{\mathbf{A}}_{\text{z}}^{{{\text{VI}}}}} \right|_{r={R_{\text{r}}}}}, \;\; {\left. {{\mathbf{H}}_{\theta }^{{\text{V}}}} \right|_{r={R_{\text{r}}}}}={\left. {{\mathbf{H}}_{\theta }^{{{\text{VI}}}}} \right|_{r={R_{\text{r}}}}}$$

On the boundary of the solution domain:

43 $${\left. {{\mathbf{A}}_{\text{z}}^{{\text{I}}}} \right|_{r={R_{{\text{sy}}}}}}=0, \;\; {\left. {{\mathbf{A}}_{\text{z}}^{{{\text{VI}}}}} \right|_{r={R_{{\text{ry}}}}}}=0$$

Taking the Fourier series in the general solution of each subdomain as a finite order and converting the Eqs. (38)−(43) into a multivariate matrix equation, as shown by Eq. (59) in the APPENDIX. Thus, all coefficient matrices (**α**^*i*^, **β**^*i*^) in Eqs. (28)-(33) can be solved, and then the MVP of each area can be solved to predict the electromagnetic performance of the in-wheel machine.

### Prediction of electromagnetic performance

Based on the obtained magnetic field solution, the electromagnetic performance of the in-wheel machine is derived. The flux linkage of the double-layer armature winding is shown below:

44 $${\varphi _{{\text{1}},i}}=\frac{{{l_{\text{a}}}}}{{{A_{\text{c}}}}}\int_{{{\theta _i} - \frac{{{b_{{\text{sa}}}}}}{2}}}^{{{\theta _i} - \frac{{{b_{{\text{sa}}}}}}{2}+d}} {\int_{{{R_{{\text{sb}}}}}}^{{{R_{\text{t}}}}} {A_{\text{z}}^{{{\text{II}}}}(r,\theta )rdrd\theta } }$$

45 $${\varphi _{2,i}}=\frac{{{l_{\text{a}}}}}{{{A_{\text{c}}}}}\int_{{{\theta _i}+\frac{{{b_{{\text{sa}}}}}}{2} - d}}^{{{\theta _i}+\frac{{{b_{{\text{sa}}}}}}{2}}} {\int_{{{R_{{\text{sb}}}}}}^{{{R_{\text{t}}}}} {A_{\text{z}}^{{{\text{II}}}}(r,\theta )rdrd\theta } }$$

46 $$\left[ \begin{gathered} {\psi _{{\text{1, A}}}} \hfill \\ {\psi _{{\text{1, B}}}} \hfill \\ {\psi _{{\text{1, C}}}} \hfill \\ \end{gathered} \right]=\frac{{{N_{\text{c}}}{C_1}}}{{{a_1}}}{\left[ {{\varphi _{1,{\text{ }}1}}{\text{ }}{\varphi _{1,{\text{ }}2}}{\text{ }} \cdots {\text{ }}{\varphi _{{\text{1, }}{Q_{\text{s}}}}}} \right]^{\text{T}}}$$

47 $$\left[ \begin{gathered} {\psi _{{\text{2, A}}}} \hfill \\ {\psi _{{\text{2, B}}}} \hfill \\ {\psi _{{\text{2, C}}}} \hfill \\ \end{gathered} \right]=\frac{{{N_{\text{c}}}{C_2}}}{{{a_1}}}{\left[ {{\varphi _{2,{\text{ }}1}}{\text{ }}{\varphi _{2,{\text{ }}2}}{\text{ }} \cdots {\text{ }}{\varphi _{2,{\text{ }}{Q_{\text{s}}}}}} \right]^{\text{T}}}$$

with $\:{\text{l}}_{\text{a}}$ being the effective axial length of the lamination, $\:{\text{a}}_{\text{1}}$ is the parallel branch number of the armature windings.

By combining with Eqs. (46) and (47), the flux linkage of three phases can be computed as

48 $$\left[ \begin{gathered} {\psi _{\text{A}}} \hfill \\ {\psi _{\text{B}}} \hfill \\ {\psi _{\text{C}}} \hfill \\ \end{gathered} \right]=\left[ \begin{gathered} {\psi _{{\text{1, A}}}} \hfill \\ {\psi _{{\text{1, B}}}} \hfill \\ {\psi _{{\text{1, C}}}} \hfill \\ \end{gathered} \right]+\left[ \begin{gathered} {\psi _{{\text{2, A}}}} \hfill \\ {\psi _{{\text{2, B}}}} \hfill \\ {\psi _{{\text{2, C}}}} \hfill \\ \end{gathered} \right]$$

The back EMF of the in-wheel machine can be calculated by taking the time derivative of the flux linkage as follows:

49 $${e_i}= - \frac{{d{\psi _i}}}{{dt}},{\text{ }}i{\text{ = A, B, C}}$$

According to the $\overrightarrow B {\mathbf{=}}\nabla \times \overrightarrow A$ principle, radial and tangential magnetic flux density expressions can be derived.

50 $${{\mathbf{B}}_r}= - j\frac{1}{r}{\mathbf{N}}{{\mathbf{A}}_\text{z}}$$

51 $${{\mathbf{B}}_\theta }= - \frac{{\partial {{\mathbf{A}}_\text{z}}}}{{\partial r}}$$

52 $${\mathbf{B}}=\sqrt {{\mathbf{B}}_{r}^{2}+{\mathbf{B}}_{\theta }^{2}}$$

Therefore, the radial- and tangential-component of the magnetic flux density in the air-gap are given by Eqs. (53) and (54). In addition, the flux densities in stator teeth/slots, slot-openings/tooth-tips, and rotor slots/teeth are given by Eqs. (112)−(117) in the **APPENDIX**.

53 $${\mathbf{B}}_{r}^{{{\text{IV}}}}= - j\frac{1}{r}{\mathbf{N}}\left[ {{{\left( {\frac{r}{{{R_{\text{m}}}}}} \right)}^{{{\mathbf{\lambda }}^{{\text{IV}}}}}}{{\mathbf{\chi }}^{{\text{IV}}}}+{{\left( {\frac{{{R_{\text{s}}}}}{r}} \right)}^{{{\mathbf{\lambda }}^{{\text{IV}}}}}}{{\mathbf{\gamma }}^{{\text{IV}}}}} \right]{e^{ - j{\mathbf{N}}\theta }}$$

54 $${\mathbf{B}}_{\theta }^{{{\text{IV}}}}= - \frac{1}{r}{{\mathbf{\lambda }}^{{\text{IV}}}}\left[ {{{\left( {\frac{r}{{{R_{\text{m}}}}}} \right)}^{{{\mathbf{\lambda }}^{{\text{IV}}}}}}{{\mathbf{\chi }}^{{\text{IV}}}} - {{\left( {\frac{{{R_{\text{s}}}}}{r}} \right)}^{{{\mathbf{\lambda }}^{{\text{IV}}}}}}{{\mathbf{\gamma }}^{{\text{IV}}}}} \right]{e^{ - j{\mathbf{N}}\theta }}$$

The accuracy of the air-gap flux density is essential because it directly affects the accuracy of the torque prediction. Maxwell’s tensor method can calculate the open-circuit cogging torque when there is no current excitation. In the same way, the electromagnetic torque can be predicted when the windings have a current source, as shown in the following formula:

55 $${T_{{\text{em}}}}=\frac{{{l_{\text{a}}}{r^2}}}{{{\mu _0}}}\int_{0}^{{2\pi }} {B_{r}^{{{\text{IV}}}}(r,\theta )B_{\theta }^{{{\text{IV}}}}(r,\theta )d\theta }$$

## Iterative algorithm

**Fig. 8:**
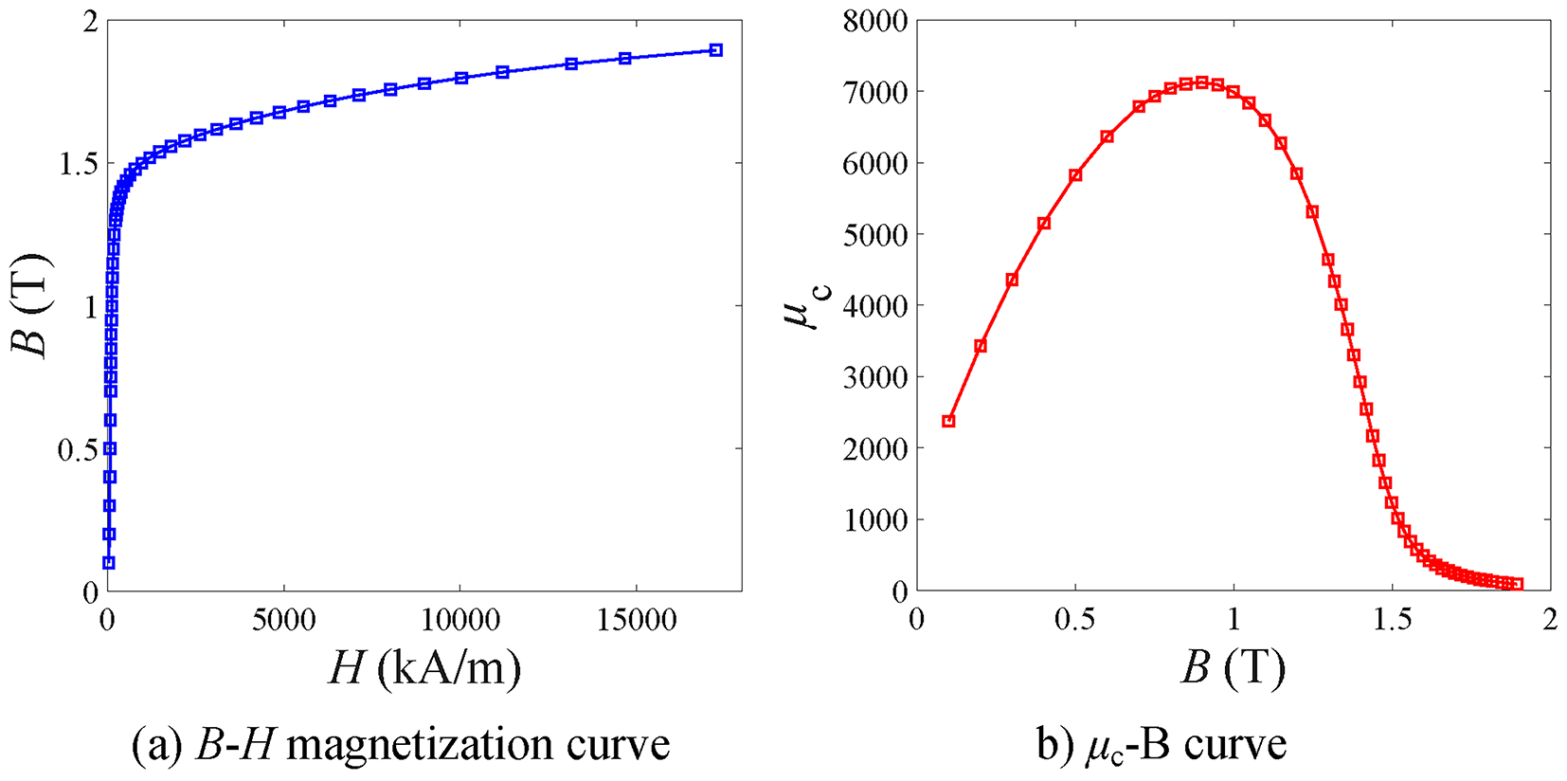
Magnetization curves of ferromagnetic material (B35AV1900).

The stator and rotor cores of the in-wheel machine are made of non-oriented silicon steel sheet B35AV1900, with nonlinear *B*-*H* magnetization characteristics shown in Fig. 8a). The relationship between relative permeability and flux density is obtained by converting the formula *µ*_c_ = *B*/(*µ*_0_*H*), as shown in Fig. 8b).

**Fig. 9:**
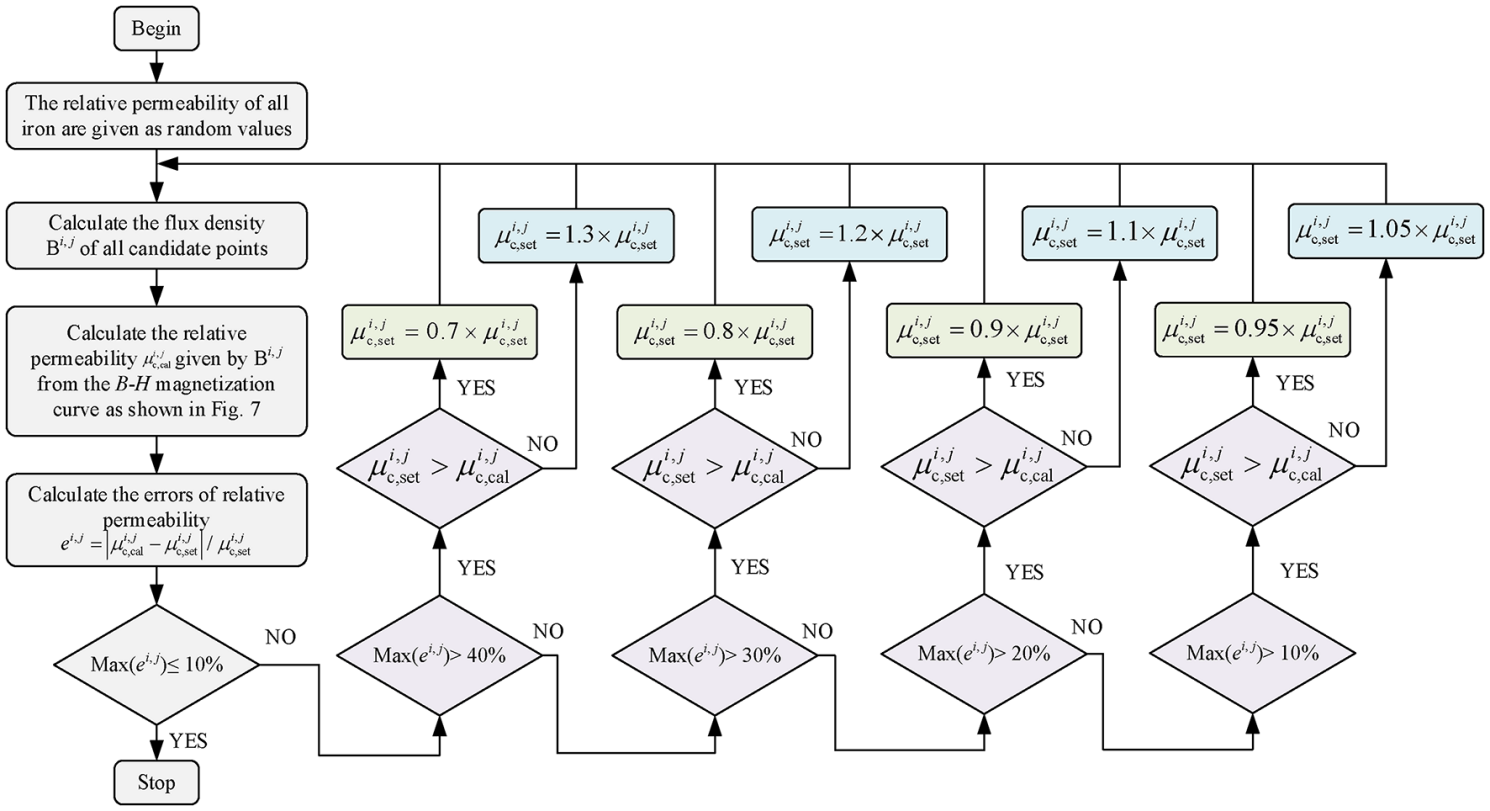
Flow chart of the iterative algorithm for calculating the permeability of the stator and rotor cores.

This paper proposes an iterative method that considers the iron’s magnetic saturation effect, and the iterative process is shown in Fig. 9. Before iteration, we selected some candidate points to approximate the magnetic density of the nearby area. On the one hand, the rotor yoke, rotor teeth, stator yoke, and stator tooth-tips are divided into several tiny areas (TAs) in the circumferential direction. The geometric center of TA is selected as candidate points, such as the magenta dots in Figs. 5 and 7, whose values approximate the permeability distribution in the TAs. On the other hand, considering the radial variation of flux density, three candidate points (green dots in Fig. 7) are selected radially on the stator teeth, and their average values are used to describe the magnetic permeability distribution in the stator teeth.

The detailed iterative process is as follows: first, initialize the relative permeability values (i.e., set value *µ*_set_) of all irons, use the proposed analytical model to predict the magnetic flux density of the stator and rotor iron cores and calculate the relative permeability value of candidate points (i.e., calculated value *µ*_cal_) through the conversion relationship shown in Fig. 8; Then, calculate the error between the set and calculated values using Eq. (56). i): If the error is greater than 40% and synergy satisfies *µ*_set_ > *µ*_cal_, multiply the *µ*_set_ by a factor of 0.7. Otherwise, if *µ*_set_ < *µ*_cal_, then the *µ*_set_ times 1.3. ii): When the error is within the range of [40%, 30%) and meets the *µ*_set_ > *µ*_cal_, the *µ*_set_ times 0.8. Otherwise, if *µ*_set_ < *µ*_cal_, then the *µ*_set_ times 1.2. iii): When the error is within the range of [30%, 20%) and meets the *µ*_set_ > *µ*_cal_, the *µ*_set_ times 0.9. Otherwise, if *µ*_set_ < *µ*_cal_, then the *µ*_set_ times 1.1. iv): When the error is within the range of [20%, 10%) and meets the *µ*_set_ > *µ*_cal_, the *µ*_set_ times 0.95. Otherwise, if *µ*_set_ < *µ*_cal_, then the *µ*_set_ times 1.05. v): The iteration ends when the error of all candidate points is less than 10%. Calculate the magnetic field distribution of the machine at this time with the current calculated value (*µ*_cal_), such as the air-gap flux density, and then obtain the electromagnetic torque value. By implication, the iterative algorithms aim to make all set and calculated values as close as possible.

56 $${e^{i,j}}=\left| {\mu _{{{\text{c,cal}}}}^{{i,j}} - \mu _{{{\text{c,set}}}}^{{i,j}}} \right|/\mu _{{{\text{c,set}}}}^{{i,j}}$$

in the formula, $\:{\text{μ}}_{\text{c,\:cal}}^{\text{i}\text{,\:}\text{j}}$ is the calculated permeability value of the *j*-th TA in Region *i*, and $\:{\text{μ}}_{\text{c,\:set}}^{\text{i}\text{,\:}\text{j}}$ is the set permeability value of the *j*-th TA in Region *i*.

**Table 1:** Main parameters of the in-wheel machine.

Symbol	Parameters	Value and unit
*P*	Rated power	10 (kW)
*V*	DC bus voltage	355.2 (V)
*I*_max_	Rated peak current	45 (A)
*ω*_*r*_	Rated speed	600 (rpm)
*N*_*c*_	Number of turns per phase wingding	224
*p*	Number of pole-pairs	16
*Q*_s_	Number of slots in stator	48
*b*_sa_	Width of stator slot	3.7822 (deg.)
*b*_oa_	Width of stator slot-opening	1.369 (deg.)
*b*_rm_	Rotor slot width angle	8.4375 (deg.)
*µ*_r_	PM relative permeability	1.05
*B*_rem_	PM remanence	1.2 (T)
*a*_1_	Number of parallel branches of windings	16
*R*_sy_	Inner radius of the stator yoke	91 (mm)
*R*_sb_	Inner radius of the stator slots	105 (mm)
*R*_t_	Outer radius of the stator slots	140 (mm)
*R*_s_	Outer radius of the stator surface	142.3 (mm)
*R*_m_	Inner radius of the PMs	143.5 (mm)
*R*_r_	Outer radius of the PMs	149.5 (mm)
*R*_ry_	Outer radius of the rotor yoke	157 (mm)
*l*_a_	Axial effective length	40 (mm)

## Analytical prediction and validation

**Fig. 10:**
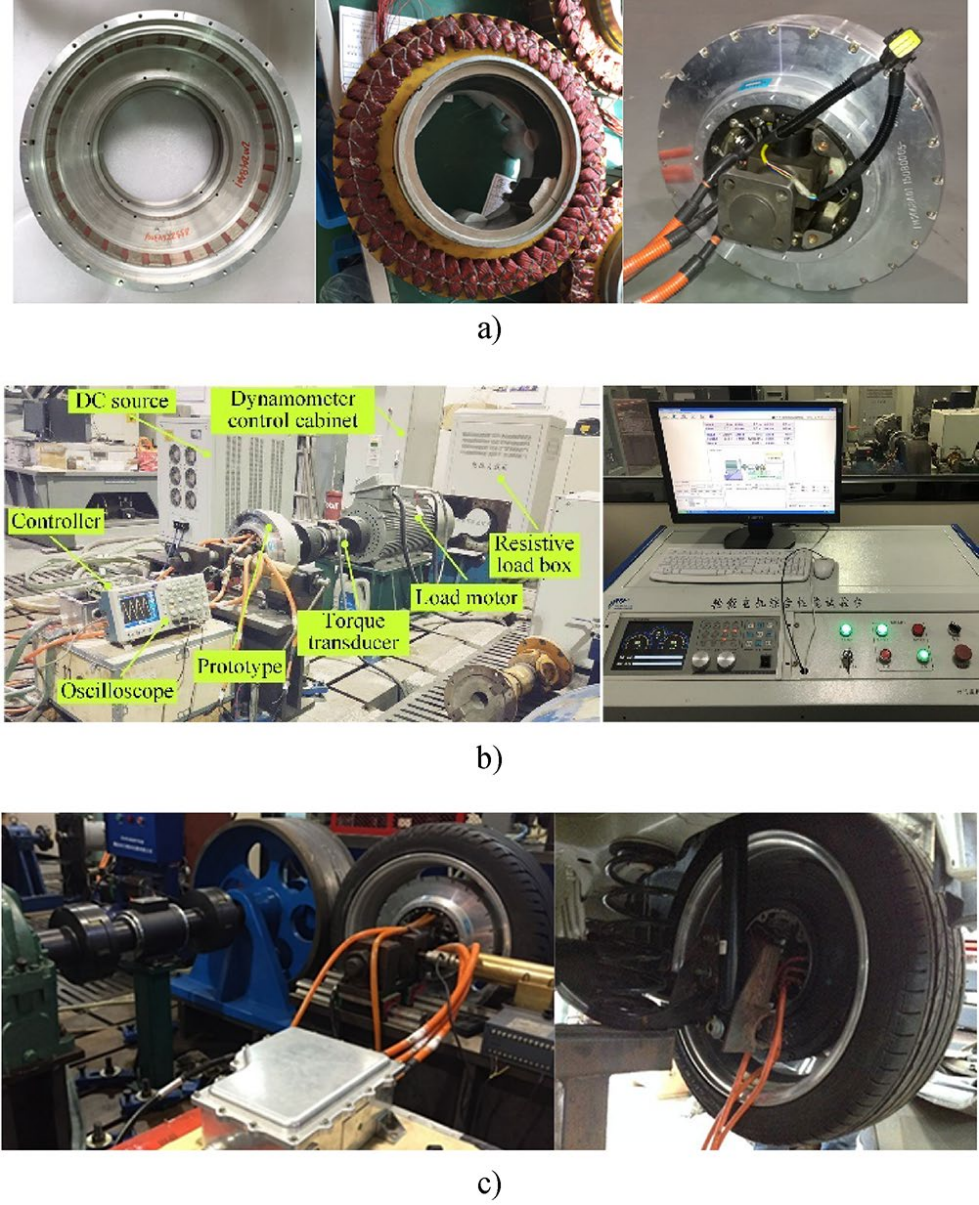
In-wheel machine prototype and electric wheel traction assembly. (a) Prototype. (b) Test bench. (c) Electric wheel traction assembly.

Take an example of a direct-drive in-wheel machine for EVs, which includes (1) an inner stator with 48 slots and fractional-slot concentrated windings, (2) an outer rotor with 16 pole pairs and surface-embedded magnets topology with radially magnetized magnets. The main parameters are listed in Table 1. We have manufactured a prototype with a rated power of 10 kW, constructed an experimental test platform with a DC bus voltage of 355.2 V, and assembled an electric wheel traction assembly, as shown in Fig. 10.

**Fig. 11:**
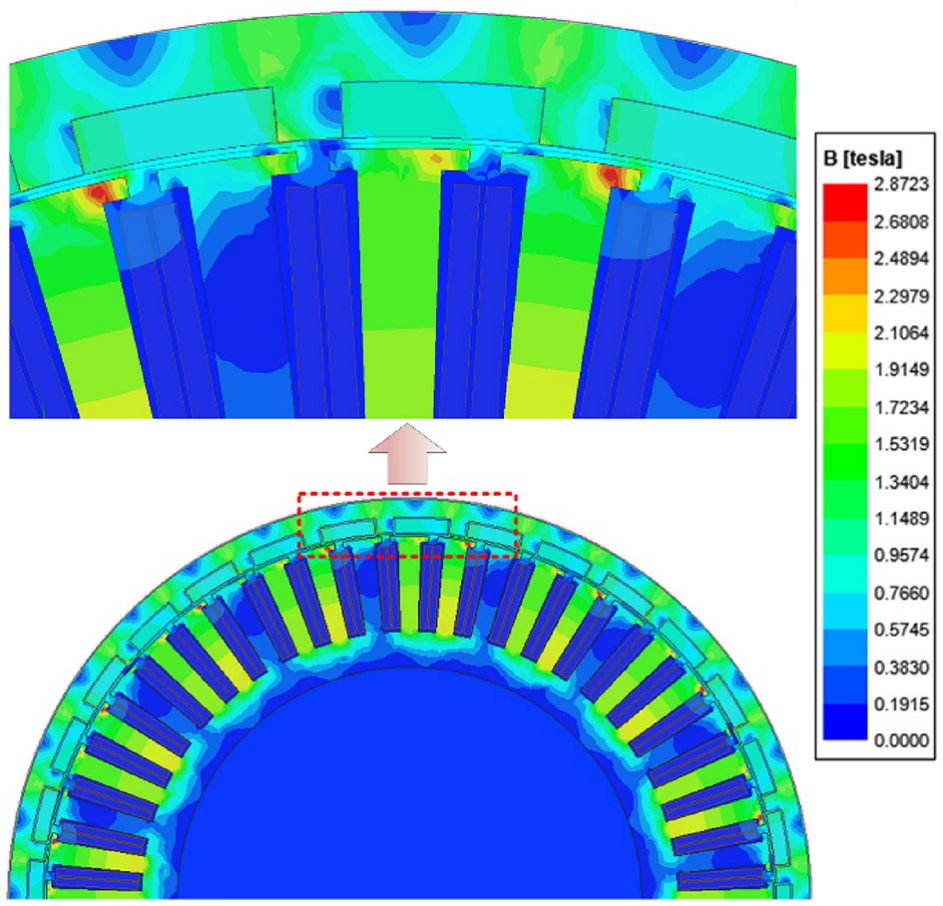
Magnetic flux lines and flux density contours simulated by finite element software (Ansys Electronics Desktop Student 2024. R2. Available from https://www.ansys.com/zh-cn/academic/students/ansys-electronics-desktop-studen.*)* under high load conditions. (*I*_max_=100 A)

To verify the proposed nonlinear analytical model’s effectiveness, we used finite element software (Ansys Electronics Desktop Student 2024. R2. Available from https://www.ansys.com/zh-cn/academic/students/ansys-electronics-desktop-studen.*)* to establish a magnetic field simulation model of the in-wheel machine considering the iron’s nonlinearity. Namely, the material characteristics of the stator and rotor iron core are set to the magnetization curve of the silicon steel sheet B35AV1900 shown in Fig. 8. The electromagnetic performance of the in-wheel machine under load conditions is simulated using the finite element software. Figure 11 is a cloud diagram of the in-wheel machine’s magnetic flux line distribution and density calculated by finite element software. It is easy to observe that magnetic saturation occurs at the stator tooth-tips under high load conditions (*I*_max_=100 A). Moreover, the flux density distribution in the tooth-tips is significantly uneven. Therefore, it is essential to subdivide the stator tooth-tips region tangentially in the analytical modeling, as expressed by Eq. (17). That is, while considering the magnetic saturation effect, it can more accurately describe the objective fact that the magnetic flux density distribution in the tooth-tips is not uniform.

**Table 2:** Parameters of the three simulation models.

Method	Parameters	Value and unit
Nonlinear FEM	Maximum element length for the coil	2.05 mm
	Maximum element length for the magnet	1.5 mm
	Maximum element length for the main domain	1.57 mm
	Surface deviation of the magnet	0.07175 mm
	Normal deviation of the magnet	5 deg
	Surface deviation of the main domain	0.0785 mm
	Normal deviation of the main domain	5 deg
SD technique	Permanent magnets	K = 30
	Air-gap	*L* = 500
	Slot-openings	*N* = 30
	Stator slots	*M* = 30
Nonlinear analytical model	Stator yoke	*N* = 400
	Stator teeth/slots	*N* = 400
	Tooth-tips/slot-opening	*N* = 400
	Air-gap	*N* = 400
	Rotor slots/teeth	*N* = 400
	Rotor yoke	*N* = 400

It must be noted that the simulation time of the nonlinear FEM is related to the length of grid elements. In contrast, the simulation time of the analytical method is associated with the maximum harmonic number of flux density and the iterative algorithm. The detailed parameter settings for the three simulation models are shown in Table 2. We use the time consumed to calculate an air-gap flux density curve (i.e., a point on the torque) as a benchmark. The SD technique takes about 4 s, the nonlinear FEM takes about 2 min and 5 s, and the proposed nonlinear model takes about 48 s. Prove that our proposed method, although slower than the SD technique, is significantly faster than the nonlinear FEM.

**Fig. 12:**
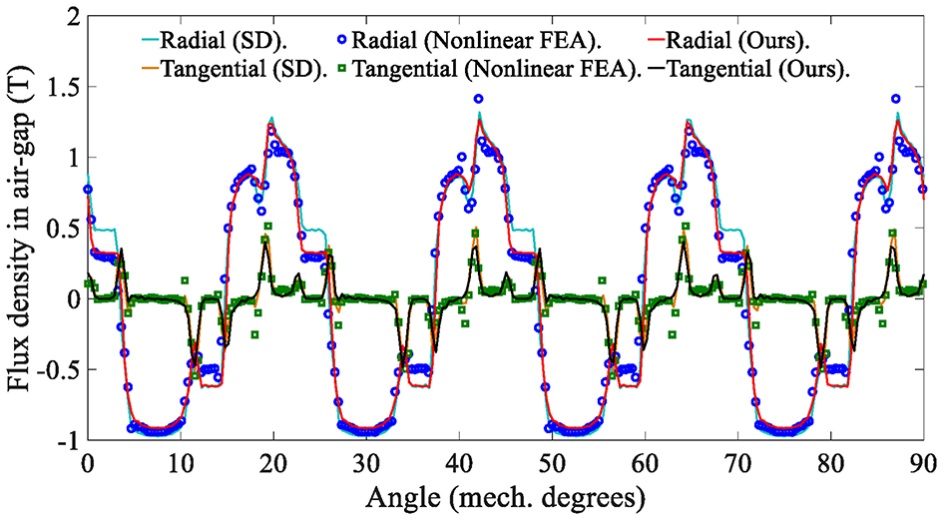
Waveform of air-gap flux density in the in-wheel machine under rated load conditions (*I*_max_ = 45 A, *r* = $\:{\text{R}}_{\text{s}}\text{+}{\text{R}}_{\text{m}}$)

**Fig. 13:**
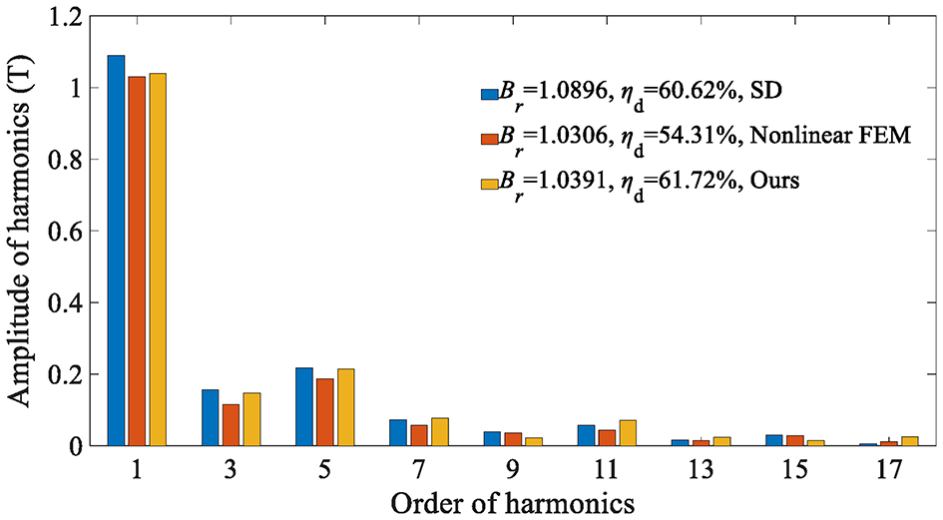
Harmonic spectrum of rated load air-gap flux density (*I*_max_ = 45 A, *r* = $\:{\text{R}}_{\text{s}}\text{+}{\text{R}}_{\text{m}}$)

The electromagnetic performance of the in-wheel machine is simulated under rated load conditions and high load conditions, respectively. For comparison, the air-gap flux density is calculated using the SD technique, the proposed analytical method, and the FEA, respectively. The maximum harmonic order of the analytical model in this study is *N* = 400. Figure 12 shows the air-gap magnetic flux density waveform under the rated operating conditions (*I*_max_=45 A), indicating that the curves of the SD technique and the nonlinear analytical model are relatively consistent. The harmonic spectrum shown in Fig. 13 confirms slight differences in the fundamental amplitude and harmonic distortion rate [Eq. (57)] between the two. This phenomenon can be explained as follows: On the one hand, when the load is not large, the magnetic saturation effect of the stator/rotor core is not apparent, so the changing trend of the curve predicted by the nonlinear analytical model is relatively consistent with that of the SD technique.

**Fig. 14:**
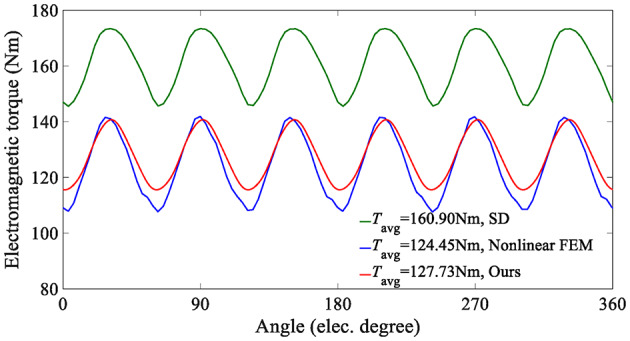
Electromagnetic torque at rated load conditions (*I*_max_ = 45 A).

On the other hand, since the SD technique makes the unreasonable assumption that the iron core is an ideal magnetic conductive material, that is, the permeability of iron is infinite, it is impossible to consider the nonlinearity of iron and describe any magnetic saturation effect. As a result, the magnetic flux density fundamental wave amplitude (1.0896T) of the SD technique is slightly higher than that of the nonlinear analytical model (1.0391T). Moreover, the overestimation of the air-gap flux density predicted by the SD technique will further lead to the overestimation of the electromagnetic torque, as shown in Fig. 14.

In addition, it can be seen that the prediction results of the nonlinear analytical model proposed in this paper are relatively consistent with those of FEA, but there are still slight errors. The reason is that the analytical model uses the magnetic flux density of the candidate points to approximate that of the TA. Namely, the flux density in the TA is considered to be uniform, while FEA has no such assumption.

57 $${\eta _{\text{d}}}{\text{=}}\sqrt {\sum\limits_{{i \ne 1}} {{{\left( {\frac{{B_{{ri}}^{{{\text{IV}}}}}}{{B_{{r1}}^{{{\text{IV}}}}}}} \right)}^2}} } \times 100{\text{\% }}\;\;i=3,\;5,\;7,\;…$$

**Fig. 15:**
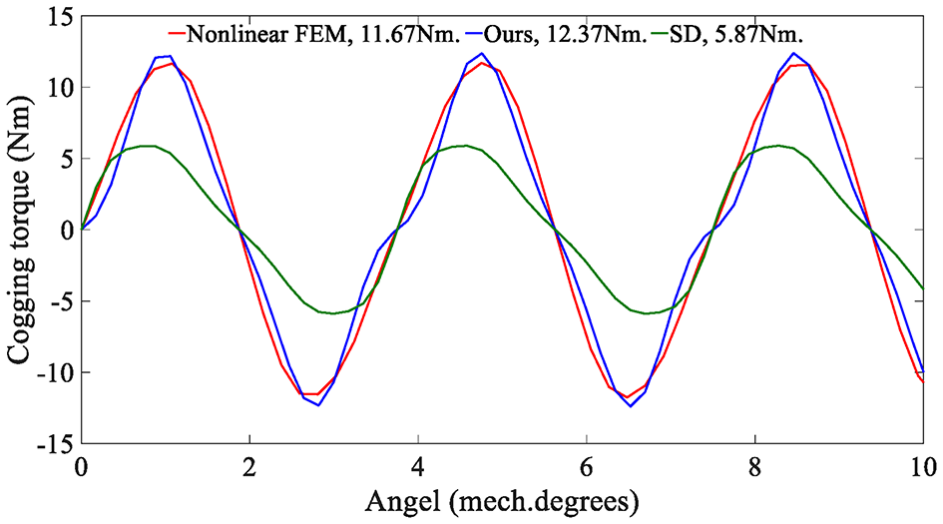
Cogging torque predicted by the analytical model and the FEA.

Figure 15 shows a comparison of cogging torque waveforms. The SD technique sets the magnetic permeability of the stator and rotor cores to infinity, which means that without reluctance, the magnetic permeability of the iron is close to perfect, resulting in smaller cogging torque. In our method, we consider the actual magnetization characteristics of iron (Fig. 8). The cogging torque curve is more accurate, as can be seen through comparison with the nonlinear FEM results.

**Fig. 16:**
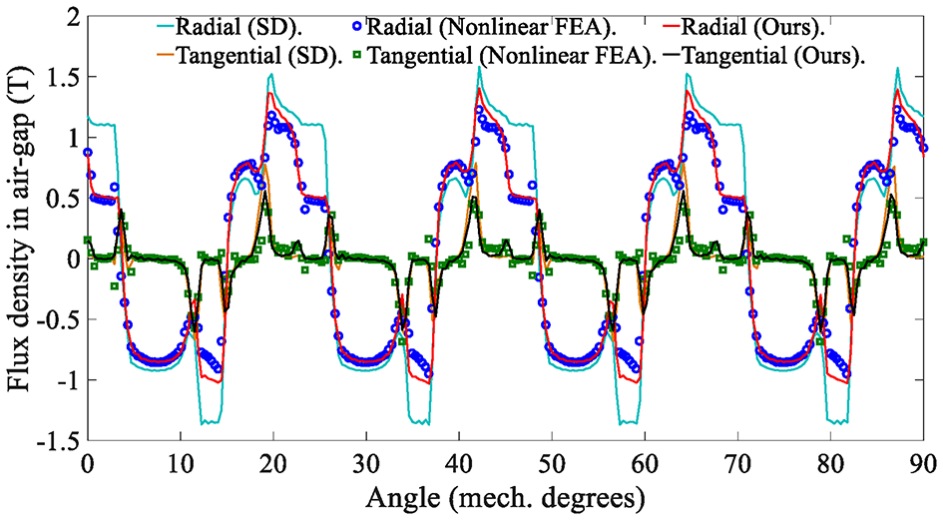
Waveform of the air-gap flux density in the in-wheel machine under high load conditions (*I*_max_ = 100 A, *r* = $\:{\text{R}}_{\text{s}}\text{+}{\text{R}}_{\text{m}}$)

**Fig. 17:**
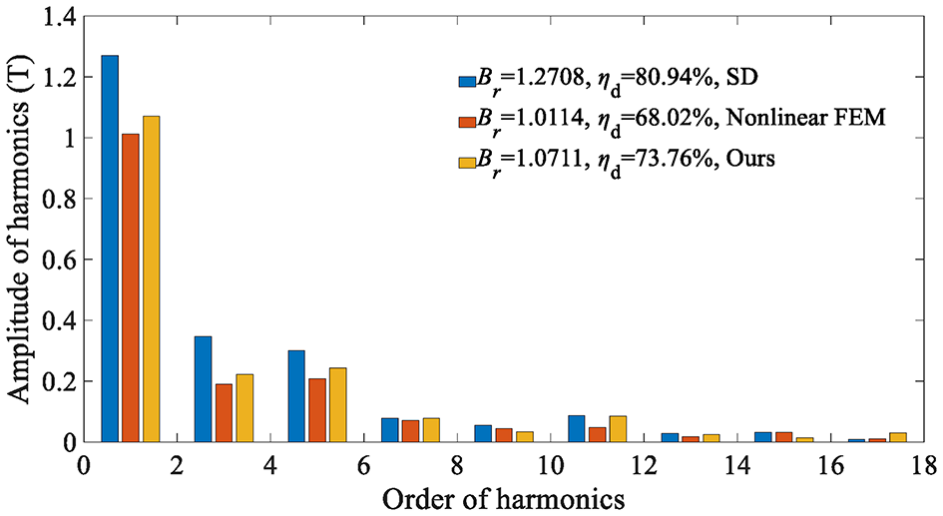
Harmonic spectrum of high load air-gap flux density (*I*_max_ = 100 A, *r* = $\:{\text{R}}_{\text{s}}\text{+}{\text{R}}_{\text{m}}$)

**Fig. 18:**
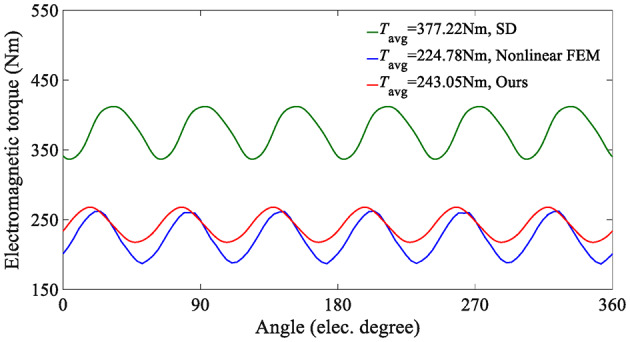
Electromagnetic torque at high load conditions (*I*_max_ = 100 A).

This paper also analyzes the electromagnetic performance of the in-wheel machine under high-load conditions. Figure 16 compares air-gap flux density curves under high-load working conditions, and Fig. 17 shows its harmonic spectrum. The flux density curve predicted by the SD technique is distorted, and its fundamental wave amplitude (1.2708T) is significantly larger than that of the nonlinear analytical model (1.0711T) and FEA (1.0114T). The essential reason, as analyzed above, is that the stator/rotor core is assumed to have infinite permeability, and the actual permeability cannot be considered, resulting in an overestimation of the air-gap flux density. As shown in Fig. 18, the magnetic saturation effect is more significant under high-load conditions. The SD technique overestimates the air-gap flux density more significantly, resulting in a significantly larger electromagnetic torque than the FEA results. In contrast, the results of the nonlinear analytical model are in good agreement with the FEA ones, proving the effectiveness of the method proposed in this paper.

In addition, the difference between the nonlinear analytical prediction and FEA results is slightly more significant than that under rated conditions. As the load increases, the magnetic saturation effect becomes more pronounced. The flux density distribution in the stator and rotor cores is more inhomogeneous. The flux density distribution in the TA represented by the candidate points will slightly increase the error.

**Fig. 19:**
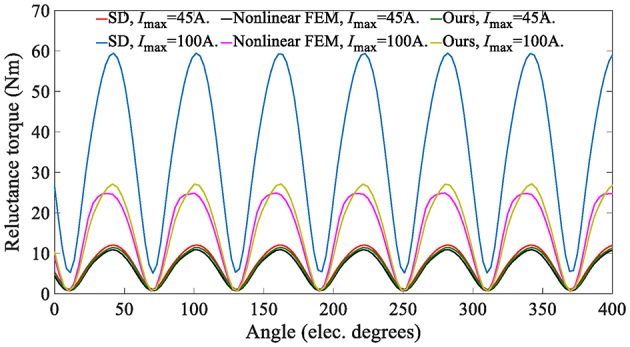
Reluctance torque predicted by the analytical model and the FEA.

When the magnetization of the magnets is 0 (***M*** = 0), the analytical model can calculate the reluctance torque. Figure 19 shows a comparison of the reluctance torque waveform. It can be seen that increasing the input current leads to a more significant reluctance torque predicted by the SD technique. In contrast, the analytical model considering magnetization characteristics is more consistent with the nonlinear FEM ones.

**Fig. 20:**
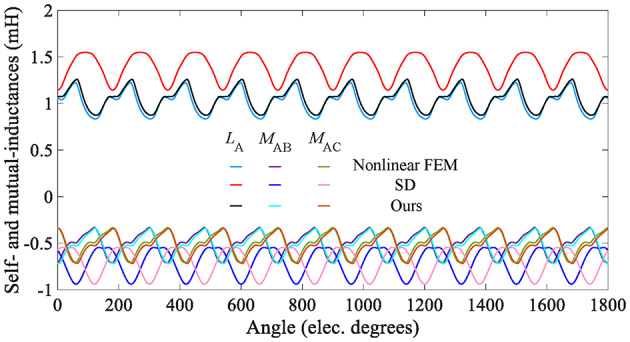
Inductance of A-phase winding changing with rotor position.

Due to the salient pole rotor of the in-wheel machine, the inductance varies with the position of the rotor. In the analytical model, if *i*_A_=100 A, *i*_B_=*i*_C_=0, *B*_*r*_=0, the self-inductance of phase A and its mutual inductance with phases B and C can be calculated, as shown in Eq. (58). Figure 20 shows the A-phase inductance curves. It can be observed that the nonlinear analytical model takes into account the actual magnetization characteristics of the stator and rotor iron core, and its inductance curve waveform is more consistent with nonlinear FEM ones. However, the SD technique cannot consider iron’s magnetization characteristics, and its waveform error is more significant.

58 $$\left[ {\begin{array}{*{20}{c}} {{L_A}} \\ {{M_{AB}}} \\ {{M_{AC}}} \end{array}} \right]=\left[ {\begin{array}{*{20}{c}} {{\psi _A}}&{{\psi _B}}&{{\psi _C}} \end{array}} \right]/{i_{\text{A}}}$$

**Fig. 21:**
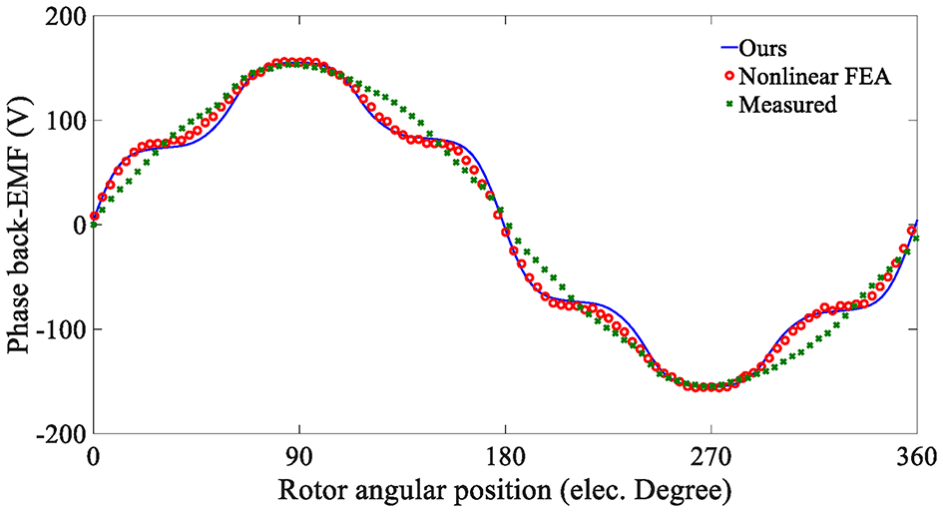
Open-circuit back-EMF predicted by the analytical model and FEA.

An open-circuit test was performed on the prototype using the experimental platform. A dynamometer measured the no-load back-EMF by dragging it to 600 r/min. As shown in Fig. 21, the back-EMF waveforms calculated by the analytical model and the nonlinear FEM are highly consistent, and the changing trends of the two waveforms are very similar. The amplitude of the measured waveform is close to the above two, but there are some errors in the trend of waveform changes. The reason is that there are errors in the shape and magnetization of the permanent magnet during the manufacturing process of the in-wheel machine, resulting in a decrease in the fifth-order harmonic content of the back-EMF waveform.

**Fig. 22:**
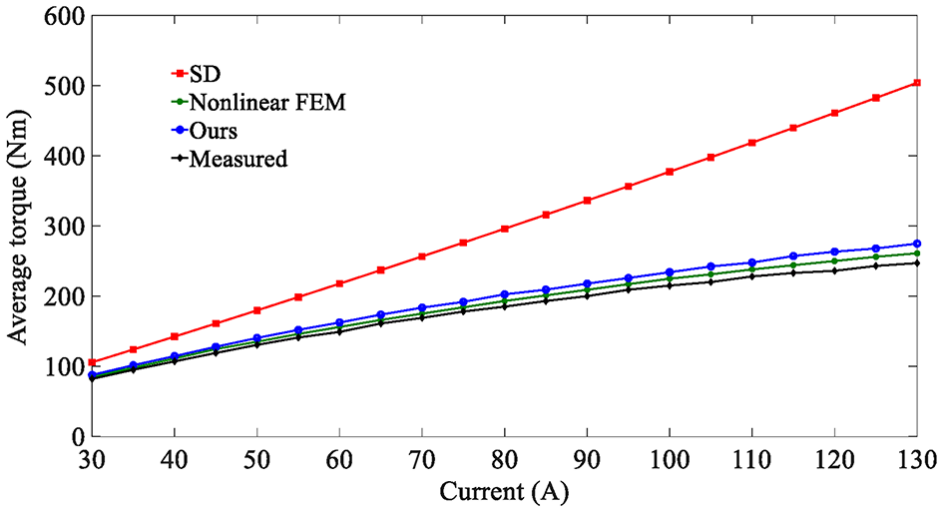
Average electronic torque versus current.

The dynamic characteristics are also carried out. Figure 22 shows that the average electromagnetic torque varies with the phase current. Due to the inability to consider magnetic saturation effects, the electromagnetic torque predicted by the SD technique increases linearly with the phase current, resulting in significant errors. The proposed analytical method considers the magnetization nonlinear characteristics of iron and can accurately predict the electromagnetic torque of the in-wheel machine under different loads. The larger the load, the more significant the magnetic saturation effect of the in-wheel machine. It has been proven that considering the magnetization nonlinearity of iron in magnetic field analytical models is critical.

Nevertheless, there are still some errors. The maximum error of our proposed nonlinear analytical model is 11.26%, and the maximum error of nonlinear FEM is 5.67%. The reason is that the analytical method and FEA put forward some specific simplifications in modeling. Furthermore, the mechanical and frictional losses in the test bench, as well as the measurement accuracy of the sensors, will also cause some errors.

## Conclusion

Based on the multi-layer model theory, this paper presents an analytical modeling method of surface-embedded in-wheel machines for EVs, considering the magnetization nonlinearity of iron. Specifically, we refined the stator tooth-tip region into three tiny areas to consider the region’s non-uniform distribution of flux density. In addition, we propose an iterative algorithm that considers the magnetic saturation effect of the machine under various loads by embedding the actual nonlinear magnetization characteristics of iron into the magnetic field solution.

The electromagnetic properties of the in-wheel machine, such as flux density, inductance, reluctance torque, cogging torque, and electromagnetic torque, are analytically predicted. The proposed nonlinear analytical model can describe the iron’s magnetic saturation effect and solve the problem of erroneous overestimation of electromagnetic torque and air-gap flux density by the conventional SD technique. It is more consistent with FEA simulation results and prototype test results, proving the effectiveness of the proposed method. It is necessary to consider the nonlinearity of iron in magnetic field analytical models of the machines.

The proposed method has wide versatility. It divides the sub-regions according to the different excitation sources and media. It is also suitable for modeling and analyzing different magnetization types and different kinds of machines, such as linear machines, spoke-type machines, induction machines, flux-switching machines, Halbach magnetization, etc.

## Electronic supplementary material

Below is the link to the electronic supplementary material.

Supplementary Material 1

## Data Availability

The data used to support the findings of this study can be obtained upon request from the corresponding author.

## References

[1] Ahmad, N. et al. Outer rotor wound field flux switching machine for in-wheel direct drive application. *IET Electr. Power Appl.* **13**(6), 757–765 (2019).

[2] Ma, C. et al. Eccentric position diagnosis of static eccentricity fault of external rotor permanent magnet synchronous motor as an in-wheel motor. *IET Electr. Power Appl.* **14**(11), 2263–2272 (2019).

[3] Zhu, Y. et al. Design and optimisation of an In-wheel switched reluctance motor for electric vehicles. *IET Intell. Transp. Sy*. **13**(1), 175–182 (2019).

[4] Zhang, H. S. et al. Analytical calculation of surface-inset PM in-wheel motors and reduction of torque ripple. *IEEE Trans. Magn.* **57**(1), 1–11 (2021).

[5] Zhu, X. et al. Design and multicondition comparison of two outer-rotor flux-switching permanent-magnet motors for in-wheel traction applications. *IEEE Trans. Ind. Electron.* **64**(8), 6137–6148 (2017).

[6] Zhang, H. et al. Comparative study on two modular spoke-type PM machines for in-wheel traction applications. *IEEE Trans. Energy Conver*. **34**(4), 2137–2147 (2019).

[7] Ngo, D., Hsieh, M. & Huynh, T. A. Torque enhancement for a novel flux intensifying PMa-SynRM using surface-inset permanent magnet. *IEEE Trans. Magn.* **55**(7), 1–8 (2019).

[8] Boroujeni, S. T., Emami, S. P. & Jalali, P. Analytical modeling of eccentric PM-inset machines with a slotless armature. *IEEE Trans. Energy Conver*. **34**(3), 1466–1474 (2019).

[9] Boroujeni, S. T. & Naghneh, H. B. Analytical modelling and prototyping a slotless surface-inset PM machine. *IET Electr. Power App*. **11**(3), 312–322 (2017).

[10] Faradonbeh, V. Z., Rahideh, A. & Markadeh, G. A. Analytical model for slotted stator brushless surface inset permanent magnet machines using virtual current theory. *IET Electr. Power Appl.* **14**(14), 2750–2761 (2020).

[11] Li, N. et al. Analysis of axial field flux-switching memory machine based on 3-D magnetic equivalent circuit network considering magnetic hysteresis. *IEEE Trans. Magn.* **55**(6), 1–4 (2019).

[12] Sun, W. et al. Electromagnetic analysis on novel rotor-segmented axial-field SRM based on dynamic magnetic equivalent circuit. *IEEE Trans. Magn.* **55**(6), 1–5 (2019).

[13] Jalali, P., Boroujeni, S. T. & Bianchi, N. Analytical modeling of slotless eccentric surface-mounted PM machines using a conformal transformation. *IEEE Trans. Energy Conver*. **32**(2), 658–666 (2017).

[14] Li, Z. et al. Analytical model of electromagnetic performance for permanent-magnet vernier machines using nonlinear exact conformal model. *IEEE Trans. Transp. Electr.* **8**(2), 2005–2014 (2022).

[15] Ullah, W., Khan, F. & Sulaiman, E. Sub-domain modelling and multi-variable optimisation of partitioned PM consequent Pole flux switching machines. *IET Electr. Power App*. **14**(8), 1360–1369 (2020).

[16] Oner, Y. et al. Analytical on-load subdomain field model of permanent magnet vernier machines. *IEEE Trans. Ind. Electron.* **63**(7), 4105–4117 (2016).

[17] Ma, C. et al. Analytical calculation of no-load magnetic field of external rotor permanent magnet brushless direct current motor used as in-wheel motor of electric vehicle. *IEEE Trans. Magn.* **54**(4), 1–6 (2018).

[18] Boroujeni, S. T. et al. Analytical investigation of the armature current influence on the torque and radial force in eccentric consequent-pole PM machines. *IET Electr. Power Appl.* **15**(4), 441–452 (2021).

[19] Zhou, Y. et al. Analytical calculation of magnetic field and cogging torque in surface-mounted permanent-magnet machines accounting for any eccentric rotor shape. *IEEE Trans. Ind. Electron.* **62**(6), 3438–3447 (2015).

[20] Ullah, W. et al. Analytical sub-domain model for magnetic field computation in segmented permanent magnet switched flux consequent Pole machine. *IEEE Access.* **9**, 3774–3783 (2021).

[21] Wu, L. J. et al. A hybrid field model for open-circuit field prediction in surface-mounted PM machines considering saturation. *IEEE Trans. Magn.* **54**(6), 1–12 (2018).

[22] Hanic, A., Zarko, D. & Hanic, Z. A novel method for no-load magnetic field analysis of saturated surface permanent-magnet machines using conformal mapping and magnetic equivalent circuits. *IEEE Trans. Energy Convers.* **31**(2), 740–749 (2016).

[23] Liang, P. et al. Analytical model of a spoke-type permanent magnet synchronous in-wheel motor with trapezoid magnet accounting for tooth saturation. *IEEE Trans. Ind. Electron.* **66**(2), 1162–1171 (2019).

[24] Sprangers, R. L. J. et al. Magnetic saturation in semi-analytical harmonic modeling for electric machine analysis. *IEEE Trans. Magn.* **52**(2), 1–10 (2016).

[25] Djelloul-Khedda, Z. et al. Semi-analytical magnetic field predicting in many structures of permanent-magnet synchronous machines considering the iron permeability. *IEEE Trans. Magn.* **54**(7), 1–21 (2018).

[26] Liang, P. et al. Calculation of the iron losses in a spoke-type permanent magnet synchronous in-wheel motor for electric vehicles by utilizing the bertotti model. *IEEE Trans. Magn.* **55**(7), 1–7 (2019).

[27] Djelloul-Khedda, Z. et al. Analytical prediction of iron-core losses in flux-modulated permanent-magnet synchronous machines. *IEEE Trans. Magn.* **55**(1), 1–12 (2019).

[28] Djelloul-Khedda, Z. et al. Nonlinear analytical prediction of magnetic field and electromagnetic performances in switched reluctance machines. *IEEE Trans. Magn.* **53**(7), 1–11 (2017).

[29] Zhang, H. et al. Analytical field model of segmented Halbach array permanent magnet machines considering iron nonlinearity. *IET Electr. Power Appl.* **15**, 717–727 (2021).

