# Supplementary material for: Analytical prediction of electromagnetic performance for surface-embedded permanent magnet in-wheel machines considering iron’s nonlinearity
Source: Sci Rep. 2024 Nov 1;14:26353. doi: 10.1038/s41598-024-77261-5 (PMC11530465; doi:10.1038/s41598-024-77261-5)
Supplement: Supplementary file 1 — Supplementary Material 1 [file 41598_2024_77261_MOESM1_ESM.docx]

# Appendix

Representing all matrix equations obtained by boundary conditions in matrix form is

$$\left[\begin{matrix} \mathbf{X}_{11} & \mathbf{X}_{12} & \mathbf{0} & \mathbf{0} & \mathbf{0} & \mathbf{0} & \mathbf{0} & \mathbf{0} & \mathbf{0} & \mathbf{0} & \mathbf{0} & \mathbf{0} \\ \mathbf{X}_{21} & \mathbf{X}_{22} & \mathbf{X}_{23} & \mathbf{X}_{24} & \mathbf{0} & \mathbf{0} & \mathbf{0} & \mathbf{0} & \mathbf{0} & \mathbf{0} & \mathbf{0} & \mathbf{0} \\ \mathbf{X}_{31} & \mathbf{X}_{32} & \mathbf{X}_{33} & \mathbf{X}_{34} & \mathbf{0} & \mathbf{0} & \mathbf{0} & \mathbf{0} & \mathbf{0} & \mathbf{0} & \mathbf{0} & \mathbf{0} \\ \mathbf{0} & \mathbf{0} & \mathbf{X}_{43} & \mathbf{X}_{44} & \mathbf{X}_{45} & \mathbf{X}_{46} & \mathbf{0} & \mathbf{0} & \mathbf{0} & \mathbf{0} & \mathbf{0} & \mathbf{0} \\ \mathbf{0} & \mathbf{0} & \mathbf{X}_{53} & \mathbf{X}_{54} & \mathbf{X}_{55} & \mathbf{X}_{56} & \mathbf{0} & \mathbf{0} & \mathbf{0} & \mathbf{0} & \mathbf{0} & \mathbf{0} \\ \mathbf{0} & \mathbf{0} & \mathbf{0} & \mathbf{0} & \mathbf{X}_{65} & \mathbf{X}_{66} & \mathbf{X}_{67} & \mathbf{X}_{68} & \mathbf{0} & \mathbf{0} & \mathbf{0} & \mathbf{0} \\ \mathbf{0} & \mathbf{0} & \mathbf{0} & \mathbf{0} & \mathbf{X}_{75} & \mathbf{X}_{76} & \mathbf{X}_{77} & \mathbf{X}_{78} & \mathbf{0} & \mathbf{0} & \mathbf{0} & \mathbf{0} \\ \mathbf{0} & \mathbf{0} & \mathbf{0} & \mathbf{0} & \mathbf{0} & \mathbf{0} & \mathbf{X}_{87} & \mathbf{X}_{88} & \mathbf{X}_{89} & \mathbf{X}_{810} & \mathbf{0} & \mathbf{0} \\ \mathbf{0} & \mathbf{0} & \mathbf{0} & \mathbf{0} & \mathbf{0} & \mathbf{0} & \mathbf{X}_{97} & \mathbf{X}_{98} & \mathbf{X}_{99} & \mathbf{X}_{910} & \mathbf{0} & \mathbf{0} \\ \mathbf{0} & \mathbf{0} & \mathbf{0} & \mathbf{0} & \mathbf{0} & \mathbf{0} & \mathbf{0} & \mathbf{0} & \mathbf{X}_{109} & \mathbf{X}_{1010} & \mathbf{X}_{1011} & \mathbf{X}_{1012} \\ \mathbf{0} & \mathbf{0} & \mathbf{0} & \mathbf{0} & \mathbf{0} & \mathbf{0} & \mathbf{0} & \mathbf{0} & \mathbf{X}_{119} & \mathbf{X}_{1110} & \mathbf{X}_{1111} & \mathbf{X}_{1112} \\ \mathbf{0} & \mathbf{0} & \mathbf{0} & \mathbf{0} & \mathbf{0} & \mathbf{0} & \mathbf{0} & \mathbf{0} & \mathbf{0} & \mathbf{0} & \mathbf{X}_{1211} & \mathbf{X}_{1212} \end{matrix}\right]\cdot\left[\begin{matrix} \mathbf{\alpha}^{\text{I}} \\ \mathbf{\beta}^{\text{I}} \\ \mathbf{\alpha}^{\text{II}} \\ \mathbf{\beta}^{\text{II}} \\ \mathbf{\alpha}^{\text{III}} \\ \mathbf{\beta}^{\text{III}} \\ \mathbf{\alpha}^{\text{IV}} \\ \mathbf{\beta}^{\text{IV}} \\ \mathbf{\alpha}^{\text{V}} \\ \mathbf{\beta}^{\text{V}} \\ \mathbf{\alpha}^{\text{VI}} \\ \mathbf{\beta}^{\text{VI}} \end{matrix}\right]= \left[\begin{matrix} \mathbf{0} \\ \mathbf{Y}_{2} \\ \mathbf{Y}_{3} \\ \mathbf{Y}_{4} \\ \mathbf{Y}_{5} \\ \mathbf{0} \\ \mathbf{0} \\ \mathbf{Y}_{8} \\ \mathbf{Y}_{9} \\ \mathbf{Y}_{10} \\ \mathbf{Y}_{11} \\ \mathbf{0} \end{matrix}\right]$$

where,

$$\mathbf{X}_{11}=\left(\frac{R_{\text{sy}}}{R_{\text{sb}}}\right)^{\mathbf{\lambda}^{\text{I}}}$$

$$\mathbf{X}_{12}=\mathbf{I}$$

$$\mathbf{X}_{21}=\mathbf{I}$$

$$\mathbf{X}_{22}=\left(\frac{R_{\text{sy}}}{R_{\text{sb}}}\right)^{\mathbf{\lambda}^{\text{I}}}$$

$$\mathbf{X}_{23}=-\mathbf{W}^{\text{II}}\left(\frac{R_{\text{sb}}}{R_{\text{t}}}\right)^{\mathbf{\lambda}^{\text{II}}}$$

$$\mathbf{X}_{24}=-\mathbf{W}^{\text{II}}$$

$$\mathbf{X}_{31}=\frac{1}{\mu_{\text{c},\theta}^{\text{I}}}\mathbf{\lambda}^{\text{I}}$$

$$\mathbf{X}_{32}=-\frac{1}{\mu_{\text{c},\theta}^{\text{I}}}\mathbf{\lambda}^{\text{I}}\left(\frac{R_{\text{sy}}}{R_{\text{sb}}}\right)^{\mathbf{\lambda}^{\text{I}}}$$

$$\mathbf{X}_{33}=-\frac{1}{\mu_{\text{c},\theta}^{\text{II}}}\mathbf{W}^{\text{II}}\mathbf{\lambda}^{\text{II}}\left(\frac{R_{\text{sb}}}{R_{\text{t}}}\right)^{\mathbf{\lambda}^{\text{II}}}$$

$$\mathbf{X}_{34}=\frac{1}{\mu_{\text{c},\theta}^{\text{II}}}\mathbf{W}^{\text{II}}\mathbf{\lambda}^{\text{II}}$$

$$\mathbf{X}_{43}=\mathbf{W}^{\text{III}}$$

$$\mathbf{X}_{44}=\mathbf{W}^{\text{II}}\left(\frac{R_{\text{sb}}}{R_{\text{t}}}\right)^{\mathbf{\lambda}^{\text{II}}}$$

$$\mathbf{X}_{45}=-\mathbf{W}^{\text{III}}\left(\frac{R_{\text{t}}}{R_{\text{s}}}\right)^{\mathbf{\lambda}^{\text{III}}}$$

$$\mathbf{X}_{46}=-\mathbf{W}^{\text{III}}$$

$$\mathbf{X}_{53}=\frac{1}{\mu_{\text{c},\theta}^{\text{II}}}\mathbf{W}^{\text{II}}\mathbf{\lambda}^{\text{II}}$$

$$\mathbf{X}_{54}=-\frac{1}{\mu_{\text{c},\theta}^{\text{II}}}\mathbf{W}^{\text{II}}\mathbf{\lambda}^{\text{II}}\left(\frac{R_{\text{sb}}}{R_{\text{t}}}\right)^{\mathbf{\lambda}^{\text{II}}}$$

$$\mathbf{X}_{55}=-\frac{1}{\mu_{\text{c},\theta}^{\text{III}}}\mathbf{W}^{\text{III}}\mathbf{\lambda}^{\text{III}}\left(\frac{R_{\text{t}}}{R_{\text{s}}}\right)^{\mathbf{\lambda}^{\text{III}}}$$

$$\mathbf{X}_{56}=\frac{1}{\mu_{\text{c},\theta}^{\text{III}}}\mathbf{W}^{\text{III}}\mathbf{\lambda}^{\text{III}}$$

$$\mathbf{X}_{65}=\mathbf{W}^{\text{III}}$$

$$\mathbf{X}_{66}=\mathbf{W}^{\text{III}}\left(\frac{R_{\text{t}}}{R_{\text{s}}}\right)^{\mathbf{\lambda}^{\text{III}}}$$

$$\mathbf{X}_{67}=-\left(\frac{R_{\text{s}}}{R_{\text{m}}}\right)^{\mathbf{\lambda}^{\text{IV}}}$$

$$\mathbf{X}_{68}=-\mathbf{I}$$

$$\mathbf{X}_{75}=\frac{1}{\mu_{\text{c},\theta}^{\text{III}}}\mathbf{W}^{\text{III}}\mathbf{\lambda}^{\text{III}}$$

$$\mathbf{X}_{76}=-\frac{1}{\mu_{\text{c},\theta}^{\text{III}}}\mathbf{W}^{\text{III}}\mathbf{\lambda}^{\text{III}}\left(\frac{R_{\text{t}}}{R_{\text{s}}}\right)^{\mathbf{\lambda}^{\text{III}}}$$

$$\mathbf{X}_{77}=-\frac{1}{\mu_{0}}\mathbf{\lambda}^{\text{IV}}\left(\frac{R_{\text{s}}}{R_{\text{m}}}\right)^{\mathbf{\lambda}^{\text{IV}}}$$

$$\mathbf{X}_{78}=\frac{1}{\mu_{0}}\mathbf{\lambda}^{\text{IV}}$$

$$\mathbf{X}_{87}=\mathbf{I}$$

$$\mathbf{X}_{88}=\left(\frac{R_{\text{s}}}{R_{\text{m}}}\right)^{\text{λ}^{\text{IV}}}$$

$$\mathbf{X}_{89}=-\mathbf{W}^{\text{V}}\left(\frac{R_{\text{m}}}{R_{\text{r}}}\right)^{\mathbf{\lambda}^{\text{V}}}$$

$$\mathbf{X}_{810}=-\mathbf{W}^{\text{V}}$$

$$\mathbf{X}_{97}=\frac{1}{\mu_{0}}\mathbf{\lambda}^{\text{IV}}$$

$$\mathbf{X}_{98}=-\frac{1}{\mu_{0}}\mathbf{\lambda}^{\text{IV}}\left(\frac{R_{\text{s}}}{R_{\text{m}}}\right)^{\mathbf{\lambda}^{\text{IV}}}$$

$$\mathbf{X}_{99}=-\frac{1}{\mathbf{\mu}_{\text{c},\theta}^{\text{V}}}\mathbf{W}^{\text{V}}\mathbf{\lambda}^{\text{V}}\left(\frac{R_{\text{m}}}{R_{\text{r}}}\right)^{\mathbf{\lambda}^{\text{V}}}$$

$$\mathbf{X}_{910}=\frac{1}{\mathbf{\mu}_{\text{c},\theta}^{\text{V}}}\mathbf{W}^{\text{V}}\mathbf{\lambda}^{\text{V}}$$

$$\mathbf{X}_{109}=\mathbf{W}^{\text{V}}$$

$$\mathbf{X}_{1010}=\mathbf{W}^{\text{V}}\left(\frac{R_{\text{m}}}{R_{\text{r}}}\right)^{\mathbf{\lambda}^{\text{V}}}$$

$$\mathbf{X}_{1011}=-\left(\frac{R_{\text{r}}}{R_{\text{ry}}}\right)^{\mathbf{\lambda}^{\text{VI}}}$$

$$\mathbf{X}_{1012}=-\mathbf{I}$$

$$\mathbf{X}_{119}=\frac{1}{\mathbf{\mu}_{\text{c},\theta}^{\text{V}}}\mathbf{W}^{\text{V}}\mathbf{\lambda}^{\text{V}}$$

$$\mathbf{X}_{1110}=-\frac{1}{\mathbf{\mu}_{\text{c},\theta}^{\text{V}}}\mathbf{W}^{\text{V}}\mathbf{\lambda}^{\text{V}}\left(\frac{R_{\text{m}}}{R_{\text{r}}}\right)^{\mathbf{\lambda}^{\text{V}}}$$

$$\mathbf{X}_{1111}=-\frac{1}{\mathbf{\mu}_{\text{c},\theta}^{\text{VI}}}\mathbf{\lambda}^{\text{VI}}\left(\frac{R_{\text{r}}}{R_{\text{ry}}}\right)^{\mathbf{\lambda}^{\text{VI}}}$$

$$\mathbf{X}_{1112}=\frac{1}{\mathbf{\mu}_{\text{c},\theta}^{\text{VI}}}\mathbf{\lambda}^{\text{VI}}$$

$$\mathbf{X}_{1211}=\mathbf{I}$$

$$\mathbf{X}_{1212}=\left(\frac{R_{\text{r}}}{R_{\text{ry}}}\right)^{\mathbf{\lambda}^{\text{VI}}}$$

$$\mathbf{Y}_{2}=R_{\text{sb}}^{2}\mathbf{\Gamma}^{\text{II}}$$

$$\mathbf{Y}_{3}=2R_{\text{sb}}^{2}\frac{1}{\mu_{\text{c},\theta}^{\text{II}}}\mathbf{\Gamma}^{\text{II}}$$

$$\mathbf{Y}_{4}=-R_{\text{t}}^{2}\mathbf{\Gamma}^{\text{II}}$$

$$\mathbf{Y}_{5}=-2R_{\text{t}}^{2}\frac{1}{\mu_{\text{c},\theta}^{\text{II}}}\mathbf{\Gamma}^{\text{II}}$$

$$\mathbf{Y}_{8}=R_{\text{m}}\mathbf{\Gamma}^{\text{V}}$$

$$\mathbf{Y}_{9}=\frac{1}{\mu_{\text{c},\theta}^{\text{V}}}R_{\text{m}}\left(\mathbf{\Gamma}^{\text{V}}+\mu_{0}\mathbf{M}_{\theta}\right)$$

$$\mathbf{Y}_{10}=-R_{\text{r}}\mathbf{\Gamma}^{\text{V}}$$

$$\mathbf{Y}_{11}=-\frac{1}{\mu_{\text{c},\theta}^{\text{V}}}R_{\text{r}}\left(\mathbf{\Gamma}^{\text{V}}+\mu_{0}\mathbf{M}_{\theta}\right)$$

The radial and tangential magnetic densities of each region can be derived from the MVP, for Region II (stator slots/teeth):

$$\mathbf{B}_{r}^{\text{II}}=-j\frac{1}{r}\mathbf{N}\left[\mathbf{\xi}^{\text{II}}\left(\frac{r}{R_{\text{t}}}\right)^{\mathbf{\lambda}^{\text{II}}}\mathbf{\chi}^{\text{II}}+\mathbf{\xi}^{\text{II}}\left(\frac{R_{\text{sb}}}{r}\right)^{\mathbf{\lambda}^{\text{II}}}\mathbf{\gamma}^{\text{II}}+r^{2}\mathbf{\Gamma}^{\text{II}}\right]$$

$$\mathbf{B}_{\theta}^{\text{II}}=-\frac{1}{r}\left[\mathbf{\xi}^{\text{II}}\mathbf{\lambda}^{\text{II}}\left(\frac{r}{R_{\text{t}}}\right)^{\mathbf{\lambda}^{\text{II}}}\mathbf{\chi}^{\text{II}}-\mathbf{\xi}^{\text{II}}\mathbf{\lambda}^{\text{II}}\left(\frac{R_{\text{sb}}}{r}\right)^{\mathbf{\lambda}^{\text{II}}}\mathbf{\gamma}^{\text{II}}+2r^{2}\mathbf{\Gamma}^{\text{II}}\right]$$

For Region III (slot-openings/tooth-tips):

$$\mathbf{B}_{r}^{\text{III}}=-j\frac{1}{r}\mathbf{N}\left[\mathbf{\xi}^{\text{III}}\left(\frac{r}{R_{\text{s}}}\right)^{\mathbf{\lambda}^{\text{III}}}\mathbf{\chi}^{\text{III}}+\mathbf{\xi}^{\text{III}}\left(\frac{R_{\text{t}}}{r}\right)^{\mathbf{\lambda}^{\text{III}}}\mathbf{\gamma}^{\text{III}}\right]$$

$$\mathbf{B}_{\theta}^{\text{III}}=-\frac{1}{r}\left[\mathbf{\xi}^{\text{III}}\mathbf{\lambda}^{\text{III}}\left(\frac{r}{R_{\text{s}}}\right)^{\mathbf{\lambda}^{\text{III}}}\mathbf{\chi}^{\text{III}}-\mathbf{\xi}^{\text{III}}\mathbf{\lambda}^{\text{III}}\left(\frac{R_{\text{t}}}{r}\right)^{\mathbf{\lambda}^{\text{III}}}\mathbf{\gamma}^{\text{III}}\right]$$

For Region V (rotor slots/teeth):

$$\mathbf{B}_{r}^{\text{V}}=-j\frac{1}{r}\mathbf{N}\left[\mathbf{\xi}^{\text{V}}\left(\frac{r}{R_{\text{r}}}\right)^{\mathbf{\lambda}^{\text{V}}}\mathbf{\chi}^{\text{V}}+\mathbf{\xi}^{\text{V}}\left(\frac{R_{\text{m}}}{r}\right)^{\mathbf{\lambda}^{\text{V}}}\mathbf{\gamma}^{\text{V}}+r\Gamma^{\text{V}}\right]$$

$$\mathbf{B}_{\theta}^{\text{V}}=-\frac{1}{r}\left[\mathbf{\xi}^{\text{V}}\mathbf{\lambda}^{\text{V}}\left(\frac{r}{R_{\text{r}}}\right)^{\mathbf{\lambda}^{\text{V}}}\mathbf{\chi}^{\text{V}}-\mathbf{\xi}^{\text{V}}\mathbf{\lambda}^{\text{IV}}\left(\frac{R_{\text{m}}}{r}\right)^{\mathbf{\lambda}^{\text{V}}}\mathbf{\gamma}^{\text{V}}+\mathbf{\Gamma}^{\text{V}}\right]$$
